# Structure-function-guided exploration of the antimicrobial peptide polybia-CP identifies activity determinants and generates synthetic therapeutic candidates

**DOI:** 10.1038/s42003-018-0224-2

**Published:** 2018-12-07

**Authors:** Marcelo D. T. Torres, Cibele N. Pedron, Yasutomi Higashikuni, Robin M. Kramer, Marlon H. Cardoso, Karen G. N. Oshiro, Octávio L. Franco, Pedro I. Silva Junior, Fernanda D. Silva, Vani X. Oliveira Junior, Timothy K. Lu, Cesar de la Fuente-Nunez

**Affiliations:** 1 Synthetic Biology Group, MIT Synthetic Biology Center; The Center for Microbiome Informatics and Therapeutics; Research Laboratory of Electronics, Department of Biological Engineering, and Department of Electrical Engineering and Computer Science, Massachusetts Institute of Technology, Cambridge, MA 02139 USA; 2 Broad Institute of MIT and Harvard, Cambridge, MA 02142 USA; 3 Centro de Ciências Naturais e Humanas, Universidade Federal do ABC, Santo André, SP 09210580 Brazil; 4 Division of Comparative Medicine, Massachusetts Institute of Technology, Cambridge, MA 02139 USA; 5 Programa de Pós-Gradução em Patologia Molecular, Faculdade de Medicina, Universidade de Brasília, Brasília, DF 70297400 Brazil; 6 Centro de Análises Proteômicas e Bioquímicas, Universidade Católica de Brasília, Brasília, DF 71966700 Brazil; 7 S-inova Biotech, Programa de Pós-Graduação em Biotecnologia, Universidade Católica Dom Bosco, Campo Grande, MS 79117010 Brazil; 8 Laboratório Especial de Toxinologia Aplicada, Instituto Butantan, São Paulo, SP 05503900 Brazil

**Keywords:** Peptides, Drug screening, Medicinal chemistry, Antimicrobials

## Abstract

Antimicrobial peptides (AMPs) constitute promising alternatives to classical antibiotics for the treatment of drug-resistant infections, which are a rapidly emerging global health challenge. However, our understanding of the structure-function relationships of AMPs is limited, and we are just beginning to rationally engineer peptides in order to develop them as therapeutics. Here, we leverage a physicochemical-guided peptide design strategy to identify specific functional hotspots in the wasp-derived AMP polybia-CP and turn this toxic peptide into a viable antimicrobial. Helical fraction, hydrophobicity, and hydrophobic moment are identified as key structural and physicochemical determinants of antimicrobial activity, utilized in combination with rational engineering to generate synthetic AMPs with therapeutic activity in a mouse model. We demonstrate that, by tuning these physicochemical parameters, it is possible to design nontoxic synthetic peptides with enhanced sub-micromolar antimicrobial potency in vitro and anti-infective activity in vivo. We present a physicochemical-guided rational design strategy to generate peptide antibiotics.

## Abstract (web summary)

Marcelo D. T. Torres et al. turn toxic wasp-derived antimicrobial peptide polybia-CP into a viable antimicrobial with therapeutic activity in a mouse model. This study demonstrates that a physicochemical property-guided rational design strategy can be used to generate peptide antibiotics.

## Introduction

Drug-resistant bacteria are a major health problem worldwide^1^. Even in developed countries such as the United States, each year ~2 million people become infected with antibiotic-resistant bacteria, resulting in at least 23,000 deaths annually^1^. Therefore, there is an urgent need to develop new therapeutics to combat drug resistance^2,3^.

Antimicrobial peptides (AMPs) represent a promising alternative to conventional antibiotics because of their potency against difficult-to-treat infections^4^, such as the ESKAPE pathogens (*Enterococcus faecium, Staphylococcus aureus, Klebsiella pneumoniae, Acinetobacter baumannii, Pseudomonas aeruginosa*, and *Enterobacter* spp.)^5^. AMPs are produced as a mechanism of defense against infections by virtually all living organisms. Some of these peptides exhibit broad-spectrum activity, targeting bacterial, fungal, parasitic, and eukaryotic cells indiscriminately. However, the biological function of AMPs may be tuned by modulating biophysical features to favor specificity, selectivity^6^, potency^7^, and other desired biological parameters to turn these molecules into novel anti-infective agents.

Despite of some obstacles, such as short serum half-life of small linear natural peptides and intrinsic bacterial resistance (i.e., membrane modifications, efflux pump and proteolytic degradation) to certain host defense peptides^8^, AMPs are a promising alternative to conventional antibiotics because of their unique diversity of peptide sequences. Their sequence space is almost unlimited, and a wide range of amino acids is available in nature^9^. Biological evolution has selected AMPs with certain sequence biases; however, even minor changes to these sequences enabled by peptide engineering may yield unprecedented biological function. The most widely studied class of AMPs comprises linear cationic amphipathic AMPs^10^, which shift from coiled to helical structures^11,12^ when the peptide comes into contact with the membranes of microorganisms.

Most AMPs act by disrupting the cytoplasmic membrane of microorganisms through several different mechanisms^13^ that do not necessarily exclude each other. Important mechanisms of action of AMPs are carpet-like, barrel stave, or toroidal pore formation^14^. Other specific or general mechanisms have been described, such as membrane thickening/thinning^15^, charged lipid clustering^16^, nucleic acids targeting^14^, anion carriers^17^, electroporation^18^, non-lytic membrane depolarization^19^, and non-bilayer intermediates^20^. However, some AMPs antimicrobial mode of action include targeting key cellular processes and metabolic pathways^21,22^ including DNA and protein synthesis^23,24^, protein folding, enzymatic activity and cell wall synthesis^25^, cell division^26^, RNA synthesis^27^, inactivation of chaperone proteins necessary for proper folding, and even targeting mitochondria^28^.

Insects, such as wasps and bees, and arachnids such as scorpions, and spiders, are rich sources of linear cationic amphipathic AMPs^9^. The South American social wasp *Polybia paulista* expresses a large variety of peptides in its venom^29^, each of which has a different biological function. Among them, the mastoparan class is a well-known group of chemotactic peptides having inflammatory and antimicrobial activities^29^. Souza et al.^30^ reported a 12-residue cationic amphipathic mastoparan-like AMP, polybia-CP (Pol-CP-NH_2_: Ile-Leu-Gly-Thr-Ile-Leu-Gly-Leu-Leu-Lys-Ser-Leu-NH_2_), which presents poor activity against Gram-negative bacteria, higher activity against Gram-positive bacteria, and toxicity towards human cells. The lower activity of Pol-CP-NH_2_ against Gram-negative bacteria was attributed to its low predicted helical content and to the presence of a hydrophilic serine residue next to its C-terminus, a residue that is not present in this position in other mastoparan-like peptides from the same wasp venom, such as protonectin and polybia-MPI^30^.

**Fig. 1:**
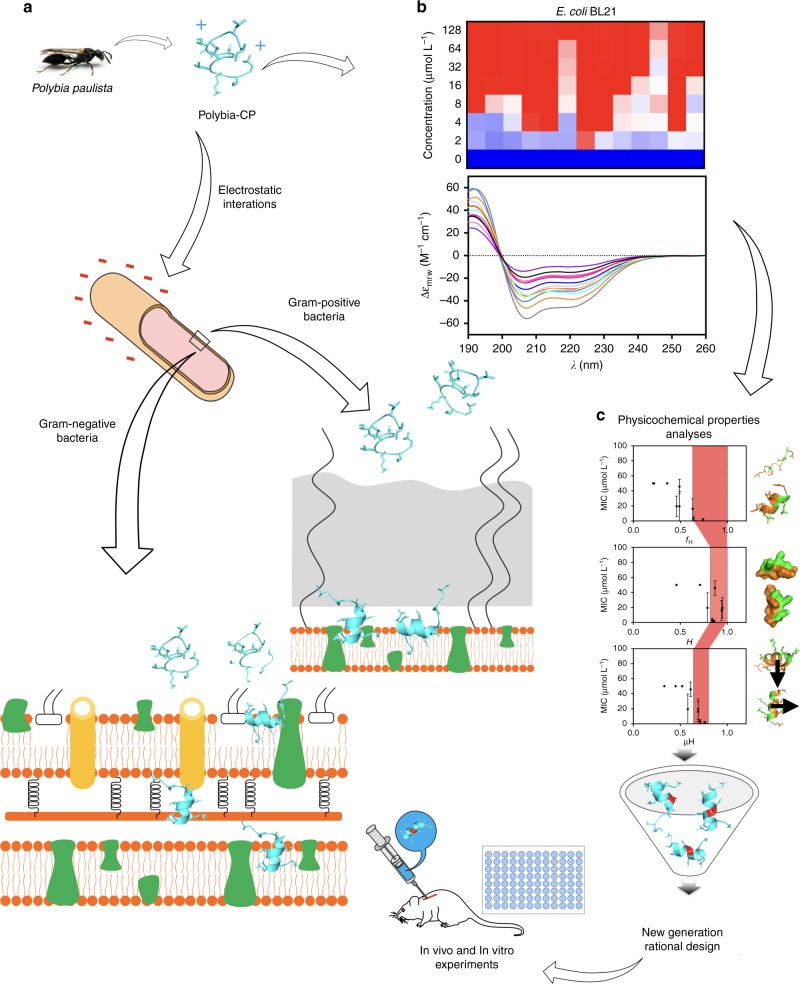
Schematic of the structure-function-guided exploration approach leveraged to generate peptide antibiotics. **a** The wasp venom derived antimicrobial peptide Polybia-CP was subjected to structure-function analysis to elucidate determinants responsible for biological activity. **b** Data from antimicrobial activity, physicochemical properties, and structure analyses was harnessed to **c** identify functional determinants and generate enhanced synthetic variants with therapeutic potential

Here, we leveraged a rational peptide design strategy aimed at tuning physicochemical features involved in structure and function such as hydrophobicity, net positive charge, and helical content, to improve the antimicrobial activity of Pol-CP-NH_2_ and generate novel peptide antibiotics (Fig. 1).

## Results

### Alanine-Scan screening of Pol-CP-NH_2_ sequence, and structural studies

The first library of peptides was designed to evaluate the role of the side chain of each residue in biological function, and to determine how substitutions to the side chain groups of each residue would alter structural and physicochemical features as compared to those of the helical wild-type peptide Pol-CP-NH_2_. Because Ala presents the smallest side chain among all-natural chiral amino acids, it was chosen to conserve the backbone size and to evaluate the effect of the native side chains on both structure and activity.

**Fig. 2:**
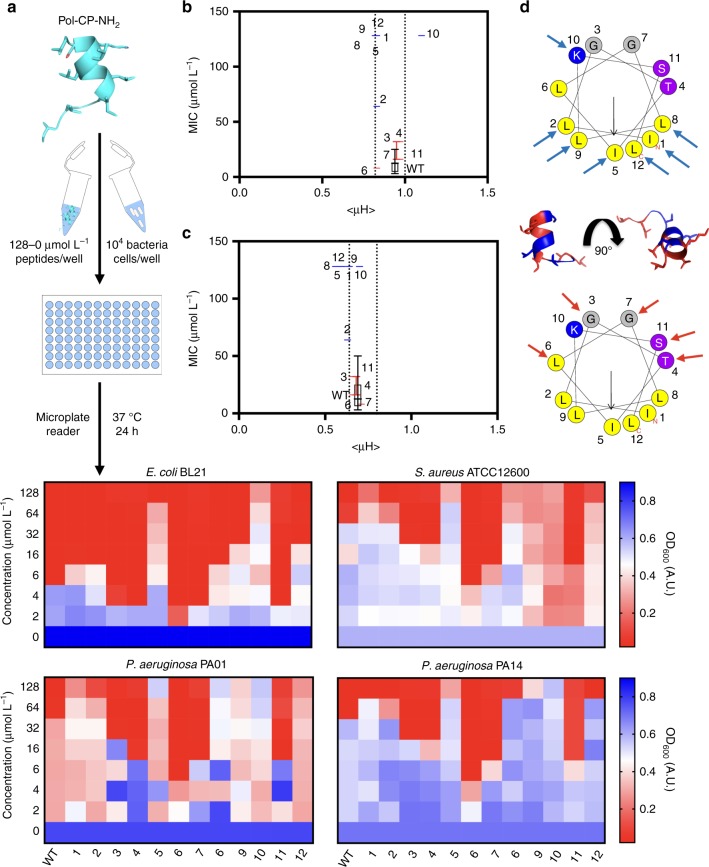
Design, physicochemical features and activity of Pol-CP-NH_2_ and Ala-scan analogs. **a** Schematic of the in vitro biological activity experimental design. Briefly, 10^4^ bacterial cells and serially diluted peptides (0–128 μmol L^−1^) were added to a 96-well plate and incubated at 37 ^o^C. One day after the exposure, the solution in each well was measured in a microplate reader (600 nm) to check inhibition of bacteria compared to the untreated controls and presented as heat maps of antimicrobial activities (μmol L^−1^) against four bacteria strains: *E. coli* strain BL21, *S. aureus* strain ATCC12600 and *P. aeruginosa* strains PA01 and PA14. Assays were performed in three independent replicates and heat map OD_600_ values are the arithmetic mean of the replicates in each condition. **b** Graph correlating MIC (μmol L^−1^) averages vs. H and **c** MIC (μmol L^−^^1^) mean vs. μH, where blue boxes represent peptides with lower activity and red boxes show peptides with higher activity compared to the wild-type, in which we can observe ranges of optimal activity in determined intervals of H and μH values. **d** Bi-dimensional helical wheels representations of the wild-type indicating positions where Ala-substitution decreased (blue arrows) and enhanced activity (red arrows) and three-dimensional representation from molecular modeling showing substitution positions in which the residues are arranged in two defined faces (hydrophobic and hydrophilic)

First, theoretical values of physicochemical features such as hydrophobicity, hydrophobic moment, and net positive charge were calculated, and helical wheels were generated using the Heliquest webserver^31^ (Table 1 and Supplementary Figure 1). Hydrophobicity values produced by the server were correlated with retention times obtained by RP-HPLC analyses (Supplementary Table 1), confirming the accuracy of the computational predictions. Next, peptides were synthesized and tested against the Gram-negative bacteria *Escherichia coli* and *Pseudomonas aeruginosa*, as well as against the Gram-positive bacterium *Staphylococcus aureus*. We obtained slightly different results from those reported by Souza et al.^30^, who described activity against Gram-positive bacteria but poor activity against Gram-negative species. The chemically synthesized wild-type peptide was active against *E. coli* [minimal inhibitory concentration (MIC) = 8.0 μmol L^−1^] and presented the same activity against *S. aureus* and both of the *P. aeruginosa* strains tested (MIC = 64.0 μmol L^−1 ^— Fig. 2a). MIC results were confirmed by colony-forming unit (CFU) counts of bacteria after one day of exposure to the peptides (Supplementary Figure 2).

**Table 1:** Theoretical physicochemical properties of interest of the wild-type and Ala-scan analogs, where *H* denotes hydrophobicity, μH is the hydrophobic moment, *q* represents the net charge and *P*/*N* is the ratio of polar/non-polar residues in the sequence

			Physicochemical Features			
Label	Peptide	Sequence	*H*	µH	*q*	*P*/*N*
WT	Pol-CP-NH_2_	ILGTILGLLKSL-NH_2_	0.94	0.69	+2	0.71
1	[Ala]^1^-Pol-CP-NH_2_	ALGTILGLLKSL-NH_2_	0.82	0.60	+2	0.71
2	[Ala]^2^-Pol-CP-NH_2_	IAGTILGLLKSL-NH_2_	0.83	0.63	+2	0.71
3	[Ala]^3^-Pol-CP-NH_2_	ILATILGLLKSL-NH_2_	0.97	0.66	+2	0.50
4	[Ala]^4^-Pol-CP-NH_2_	ILGAILGLLKSL-NH_2_	0.95	0.68	+2	0.50
5	[Ala]^5^-Pol-CP-NH_2_	ILGTALGLLKSL-NH_2_	0.82	0.56	+2	0.71
6	[Ala]^6^-Pol-CP-NH_2_	ILGTIAGLLKSL-NH_2_	0.83	0.71	+2	0.71
7	[Ala]^7^-Pol-CP-NH_2_	ILGTILALLKSL-NH_2_	0.97	0.66	+2	0.50
8	[Ala]^8^-Pol-CP-NH_2_	ILGTILGALKSL-NH_2_	0.83	0.64	+2	0.71
9	[Ala]^9^-Pol-CP-NH_2_	ILGTILGLAKSL-NH_2_	0.83	0.70	+2	0.71
10	[Ala]^10^-Pol-CP-NH_2_	ILGTILGLLASL-NH_2_	1.10	0.60	+1	0.50
11	[Ala]^11^-Pol-CP-NH_2_	ILGTILGLLKAL-NH_2_	0.97	0.67	+2	0.50
12	[Ala]^12^-Pol-CP-NH_2_	ILGTILGLLKSA-NH_2_	0.83	0.58	+2	0.71

Substitution analysis with Ala revealed that, when Ile at position 5 and, independently, Lys at position 10 were substituted, the most drastic decreases in antimicrobial activity were observed against both Gram-positive and Gram-negative bacteria (Fig. 2a), indicating that residues [Ile]^5^ and [Lys]^10^ are important for the biological activity of these peptides. Conversely, single Ala-substitutions of [Gly]^7^ and [Ser]^11^ led to a pronounced enhancement in antimicrobial activity (Fig. 2a). These assays further enabled the identification of a functional hotspot range determined by hydrophobicity and hydrophobic moment values for optimal antimicrobial activity of our Pol-CP-NH_2_ variants (blue boxes, Fig. 2b, c). In addition, modifications to the hydrophobic face of the wild-type peptide (blue arrows, Fig. 2d) led to decreased antimicrobial function, with the exception of [Leu]^6^, which is at the interface between the hydrophobic and hydrophilic faces of the helical wheel and one helical step from the charged residue [Lys]^10^ (Fig. 2a). Substitution of [Leu]^6^ may lead to destabilization of the helix and likely does not affect the antimicrobial activity by not abruptly changing the amphipathic balance. On the other hand, all changes made to the hydrophilic face led to increased antimicrobial activity, except with the positively charged residue [Lys]^10^ (Fig. 2a).

**Fig. 3:**
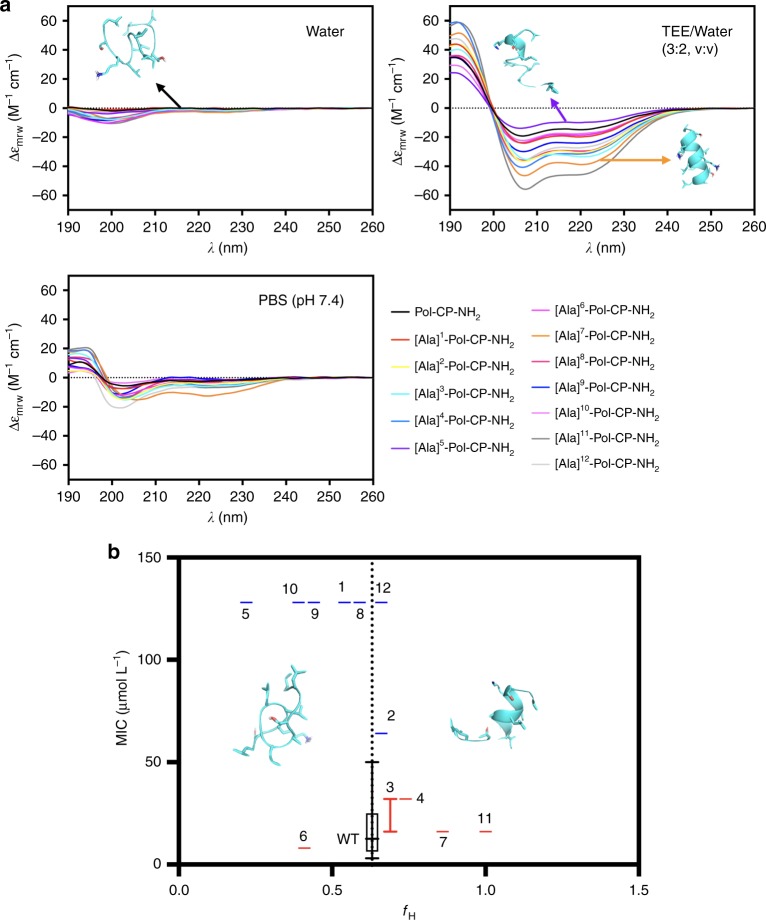
Physicochemical features and structure of Pol-CP-NH_2_ and Ala-scan analogs. **a** Circular dichroism spectra of Pol-CP-NH_2_ and Ala-scan derivatives at 50 µmol L^−1^ in water, PBS (pH 7.4) and TFE/Water (3:2, v/v) showing peptides transition from unstructured in water to helically structured in TFE/water. Circular dichroism spectra were recorded after four accumulations at 20 ^o^C, using a 1 mm path length quartz cell, between 260 and 190 nm at 50 nm min^-1^, with a bandwidth of 0.5 nm. **b** MIC (µmol L^−1^) average for each peptide against the first set of bacteria (*E. coli* BL21, *P. aeruginosa* PA01 and PA14, and *S. aureus* ATCC12600) in three independent replicates vs. *f*_H_ in TFE/Water solution, where blue boxes represent peptides with lower activity and red boxes show peptides with higher activity compared to the wild-type. Optimal activity is reached in most of the cases for *f*_H_ values higher than the wild-type

To further investigate the effect of side chains on the structure of Pol-CP-NH_2_, we performed circular dichroism spectroscopy measurements, a rapid and widely used technique for analyzing peptides secondary structure^32^. In particular, we were interested in investigating structure transitions, such as helix-coil transitions^11^, a well-known characteristic of AMPs^33–37^, usually observed from water or polar media to hydrophobic or helical inducer-media. For this reason, circular dichroism measurements of Ala-scan derivatives were initially performed in three conditions: water, PBS buffer (pH 7.4), and trifluoroethanol (TFE) in water (3:2; v-v). PBS buffer was chosen to evaluate the effects of peptide exposure to ions at neutral pH (7.4), due to its low absorbance of polarized light at the wavelength range analyzed (195–260 nm). TFE/water solution is widely used in studies of peptide structure as it promotes the formation of helical structures and stability^38,39^. As expected, the peptides presented an undefined secondary structure in water and a secondary structure with small helical fractions in PBS buffer (saline environment). In contrast, in the presence of TFE/water solution, the peptides tended to display a helical structure (Fig. 3a and Supplementary Figure 3), a behavior expected for small cationic amphipathic peptides^39^ and consistent with Lifson-Roig’s helix-coil transition theory^11^. Most of the derivatives that presented a higher helical fraction than the wild-type (Table 2) tended to be more active than the wild-type molecule against both Gram-positive and Gram-negative bacteria (Fig. 2a). Thus, the results of the present investigation reveal some correlation between the structural (Fig. 3b) and physicochemical features (Fig. 2b, c) with antimicrobial activity, thereby opening the door to rational design strategies. The exception was [Ala]^6^-Pol-CP-NH_2_, in which the Ala-substitution led to a lower helical fraction of the peptide in helical inducer medium, and preserved the antimicrobial activity of the peptide. This discrepancy might be explained by the higher helical propensity of the Leu residue when compared to the Ala residue^40^. The Ala-substitution did not compromise the activity of the peptide since it did not lead to changes in the geometrical distribution of the other residues of the sequence maintaining the activity of this. In order to test this possibility, we next engineered novel Pol-CP-NH_2_ analogs to further validate the optimal functional hotspot ranges observed (Fig. 2b, c).

**Table 2:** Helical fraction (*f*_H_) of the Pol-CP-NH_2_ and Ala-scan derivatives in each condition analyzed

		*f*_H_		
Label	Peptide	Water	PBS	TFE:Water (3:2)
WT	Pol-CP-NH_2_	0.05	0.10	0.63
1	[Ala]^1^-Pol-CP-NH_2_	0.01	0.06	0.44
2	[Ala]^2^-Pol-CP-NH_2_	0.10	0.10	0.67
3	[Ala]^3^-Pol-CP-NH_2_	0.03	0.14	0.74
4	[Ala]^4^-Pol-CP-NH_2_	0.04	0.13	0.69
5	[Ala]^5^-Pol-CP-NH_2_	0.02	0.04	0.22
6	[Ala]^6^-Pol-CP-NH_2_	0.04	0.08	0.41
7	[Ala]^7^-Pol-CP-NH_2_	0.07	0.28	0.86
8	[Ala]^8^-Pol-CP-NH_2_	0.02	0.06	0.66
9	[Ala]^9^-Pol-CP-NH_2_	0.02	0.02	0.54
10	[Ala]^10^-Pol-CP-NH_2_	0.01	0.04	0.39
11	[Ala]^11^-Pol-CP-NH_2_	0.05	0.16	1.00
12	[Ala]^12^-Pol-CP-NH_2_	0.03	0.14	0.59

Molecular dynamics simulations of the peptides were performed in water and in 60% TFE/water solution (v/v). The simulations were performed to better understand the behavior of the three-dimensional theoretical structure (Supplementary Figure 3) of some of the Ala-scan analogs that presented different antimicrobial activities (Fig. 2; Table 1) and structural tendencies (Fig. 3; Table 2). After 100 ns of molecular dynamics simulations in both media (Supplementary Figure 3B), all analogs were found to be highly stable, as indicated by the low values of root mean square deviation, which is the measure of the average distance between the atoms of the superimposed peptides during the simulation time^41,42^, and root mean square fluctuation obtained (Supplementary Figure 3A), which is a measure of the deviation of the position of a particle with respect to a reference position over the simulation time^41,42^. In water, all the peptides were mostly unstructured after 100 ns, while in the TFE/water solution [Ala]^7^-Pol-CP-NH_2_ and [Ala]^10^-Pol-CP-NH_2_ tended to display a well-defined helical structure, and [Ala]^5^-Pol-CP-NH_2_ exhibited a less-defined helical structure. In addition, the radius of gyration was maintained over time (Supplementary Figure 3A), indicating that the molecules did not bend in both media and remained helical or coiled. These parameters, in addition to the three-dimensional structures observed throughout the simulation (Supplementary Figure 3B), revealed that, when substitutions are made to the hydrophilic face of Pol-CP-NH_2_, the analogs appear to be less highly structured (i.e., random-coiled) in water, but helical in TFE/water solution. When changes were made to the hydrophobic core of the molecule, the tendency towards adopting a helical structure was maintained in TFE/water and sometimes decreased in the same medium (Supplementary Figure 3B), consistent with our circular dichroism spectra results (Fig. 3a). Samples in TFE/water had similar root mean square deviation, root mean square fluctuation, and radius of gyration values (Supplementary Figure 3A) in comparison with simulations in water alone, indicating the structural stability of this family of peptides (Supplementary Figure 3B).

### Rationally designed Pol-CP-NH_2_ derivatives

Most wasp venom peptides present conserved motifs in their sequences, e.g., Pol-CP-NH_2_ is similar to protonectin (Ile-Leu-Gly-Thr-Ile-Leu-Gly-Leu-Leu-Lys-Gly-Leu-NH_2_)^43^. Therefore, to design the next generation of Pol-CP-NH_2_ derivatives, we generated single-substitution mutants to elucidate structure–function relationships and to identify physicochemical activity determinants (Table 3 and Supplementary Figure 4). The positions selected for the substitutions were chosen based on the Ala-scan screening results obtained (Fig. 2; Table 1), and modifications were rationally proposed by fine-tuning select physicochemical functional determinants (i.e., hydrophobicity, hydrophobic moment, and helical propensity).

**Table 3:** Theoretical physicochemical properties of interest of the wild-type and the newly designed derivatives, where *H* denotes hydrophobicity, μH is the hydrophobic moment, *q* represents the net charge and *P*/*N* is the ratio of polar/non-polar residues in the sequence. Modifications in italic indicate insertion of residues that led to increased net positive charge, in bold insertion of residues that led to decreased net positive charge, and underlined, insertion of hydrophobic/aliphatic residues

			Physicochemical features			
Peptide	Name	Sequence	*H*	µH	*q*	*P/N*
WT	Pol-CP-NH_2_	ILGTILGLLKSL-NH_2_	0.94	0.69	+2	0.71
13	[Leu]^5^-[Lys]^9^-Pol-CP-NH_2_	ILGTLLGL*K*KSL-NH_2_	0.71	0.52	+3	0.50
14	[Lys]^5^-Pol-CP-NH_2_	ILGT*K*LGLLKSL-NH_2_	0.71	0.45	+3	0.50
15	[Lys]^4^-Pol-CP-NH_2_	ILG*K*ILGLLKSL-NH_2_	0.84	0.71	+3	0.71
16	[Lys]^7^-Pol-CP-NH_2_	ILGTIL*K*LLKSL-NH_2_	0.86	0.76	+3	0.71
17	[Phe]^9^-Pol-CP-NH_2_	ILGTILGLFKSL-NH_2_	0.95	0.69	+2	0.71
18	Des[Leu]^12^-Pol-CP-NH_2_	ILGTILGLLKS-NH_2_	0.87	0.61	+2	0.83
19	[Glu]^3^-[Lys]^5^-[Glu]^12^-Pol-CP-NH_2_	IL**E**T*K*LGLLKS**E**-NH_2_	0.46	0.33	+1	0.50
20	[Gly]^1^-Pol-CP-NH_2_	GLGTILGLLKSL-NH_2_	0.79	0.58	+2	0.50

Lys was used as a basic residue to increase net positivet charge into the sequence^40^, its less hydrophobic and more flexible side chain compared to Arg side chain, confers lower propensity to cytotoxicity to the peptide^44,45^. Moreover, Lys residues are more frequent than Arg residues in naturally occurring wasp venom peptides^29^.

Leu and Phe residues were chosen to incorporate hydrophobicity into the sequence. Leu residue substitution leads to lower amount of energy required for the peptides to adopt helical structure^40^, which in most cases, favors antimicrobial activity (Figs. 1,2 and Table 1). In addition, Leu residues occur at higher frequency in wasp venom peptide sequences^29^ than other aliphatic residues. On the other hand, the Phe residue was chosen because of its bulky effect and higher hydrophobicity values^45^ compared to large aliphatic residues, making it possible to evaluate the effect of adding an aromatic residue to the hydrophobic face on structure and biological function. Additionally, unlike the hydrophobic Trp residue, Phe residues are not major components of cell-penetrating peptides^46^. However, cell penetrating peptides with high content of aromatic residues are typically cytotoxic, and are therefore better candidates for design improvements. Taking these guidelines into account (Supplementary Figure 5 and Supplementary Table 2), we generated a second-generation peptide library that aimed to unveil further structure-activity relationships (Table 3).

**Fig. 4:**
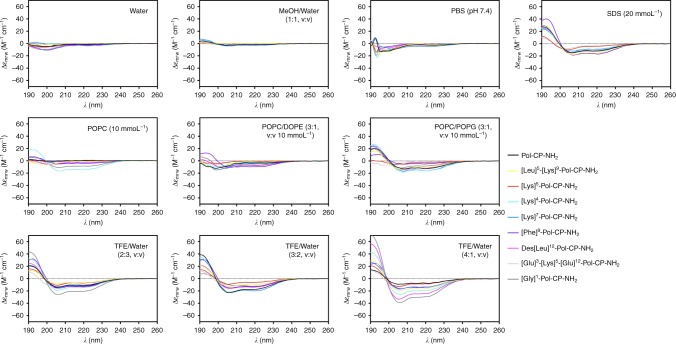
Physicochemical features and structure of Pol-CP-NH_2_ and second-generation analogs. Circular dichroism spectra of the peptides at 50 µmol L^−1^ in water, MeOH/Water (1:1, v/v), PBS (pH 7.4), POPC (10 mmol L^−1^), POPC:DOPE (3:1, 10 mmol L^−1^), POPC:POPG (3:1, 10 mmol L^−1^), SDS (20 mmol L^−1^), TFE/Water (2:3, 3:2, 4:1, v/v) showing peptides transition from unstructured in water to helically structured in TFE/water. Circular dichroism spectra were recorded after four accumulations at 20 ^o^C, using a 1 mm path length quartz cell, between 260 and 190 nm at 50 nm min^−1^, with a bandwidth of 0.5 nm

First, the effects of each substitution on the theoretical values of specific physicochemical features were assessed (Table 3) and the structures of these new analogs were analyzed by circular dichroism in ten different media (Fig. 4) that mimicked potential environments encountered by peptides, such as water, saline, and hydrophobic environments. Bacterial membranes are composed of anionic lipids, such as phosphatidylglycerol (PG), and zwitterionic lipids, such as phosphatidylethanolamine (PE), which are important for membrane organization. The lipid composition varies among bacteria, e.g., the cell membrane of Gram-negative bacteria presents a higher content of PE than that of Gram-positives; on the other hand, Gram-positive membranes are composed of higher levels of anionic lipids (e.g., PG)^47^. In order to mimic these membrane environments^48–50^, one micelle and three vesicle formulations were prepared: SDS (20 mmol L^−1^), POPC (10 mmol L^−1^), and POPC:DOPE (3:1, mol:mol, 10 mmol L^−1^), zwitterionic lipids, and POPC:POPG (3:1, mol:mol, 10 mmol L^−1^), a negatively charged unilamellar vesicle. The structure of the peptides in TFE/water solutions, which are well-known peptide helix inducers, was also analyzed^39^. The helical fraction values obtained in all circular dichroism spectra analyses are shown in Table 4. The most active peptides are inside the hotspot predicted with the Ala-Scan analogs previously. Pol-CP-NH_2_ and analogs did not tend to form β-conformations in the presence of methanol, which is known as a β-structure promoter^51^. Peptides presented higher helical fraction values when in contact with negatively charged and zwitterionic vesicles than when in contact with positively charged vesicles. The exceptions were the most hydrophobic analog, [Phe]^9^-Pol-CP-NH_2_, and [Gly]^1^-Pol-CP-NH_2_ that presented the same helical fraction values in contact with negatively and positively charged vesicles. Interestingly, the analog [Gly]^1^-Pol-CP-NH_2_ presented high helical fraction values when in contact with zwitterionic vesicles even with the introduction of a Gly residue that does not show high helical propensity. The antimicrobial activity of [Gly]^1^-Pol-CP-NH_2_ was similar to the most active analogs with higher positive net charge (Table 4).

**Table 4:** Helical fraction (*f*_H_) of the wild-type and second-generation of peptides in each condition analyzed

						*f*_H_					
Peptide	Name	Water	PBS	TFE/Water (2:3)	TFE/Water (3:2)	TFE/Water (4:1)	SDS	POPC	POPC/POPG (3:1)	POPC/DOPE (3:1)	MeOH/Water (1:1)
WT	Pol-CP-NH_2_	0.05	0.10	0.40	0.63	0.29	0.43	0	0.45	0.12	0.06
13	[Leu]^5^-[Lys]^9^-Pol-CP-NH_2_	0.03	0.04	0.23	0.36	0.58	0.55	0.04	0.38	0.07	0
14	[Lys]^5^-Pol-CP-NH_2_	0.03	0.04	0.25	0.22	0.32	0.15	0	0.08	0.04	0
15	[Lys]^4^-Pol-CP-NH_2_	0.03	0.10	0.51	0.64	0.69	0.32	0.53	0.57	0.09	0.06
16	[Lys]^7^-Pol-CP-NH_2_	0.14	0.18	0.37	0.74	0.51	0.36	0.02	0.58	0.16	0.10
17	[Phe]^9^-Pol-CP-NH_2_	0.08	0.04	0.49	0.46	0.56	0.61	0.15	0.15	0.34	0
18	Des[Leu]^12^-Pol-CP-NH_2_	0.12	0.03	0.44	0.49	0.88	0.33	0.10	0.42	0.25	0
19	[Glu]^3^-[Lys]^5^-[Glu]^12^-Pol-CP-NH_2_	0.05	0.03	0.24	0.21	0.31	0.13	0	0.04	0.03	0.02
20	[Gly]^1^-Pol-CP-NH_2_	0.11	0.07	0.74	0.49	0.99	0.45	0.33	0.28	0.28	0.09

**Fig. 5:**
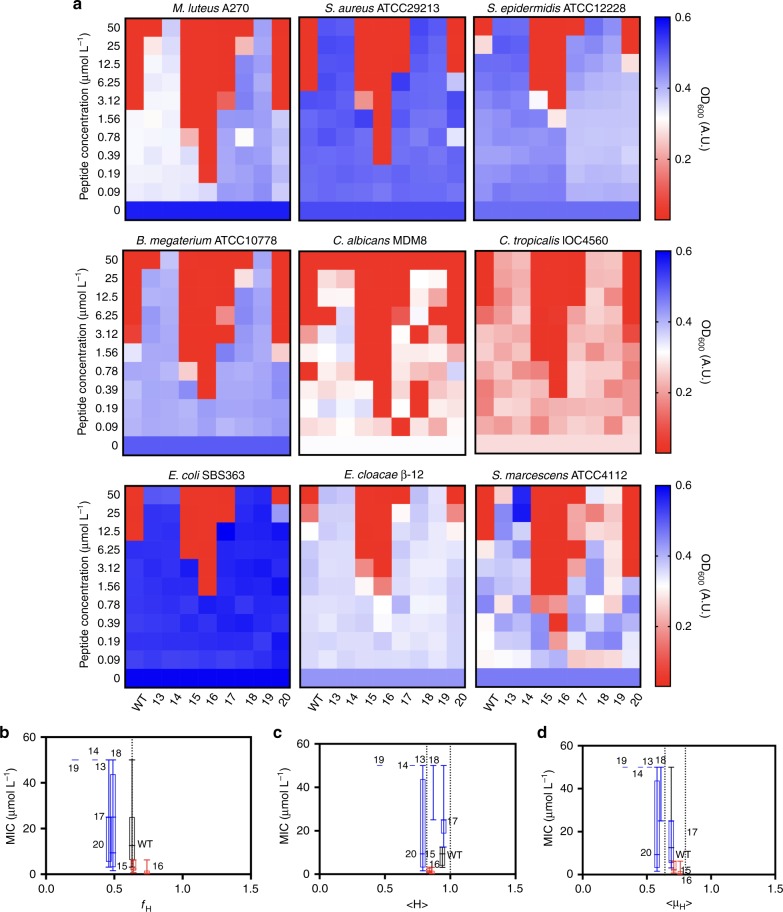
Antimicrobial activity of second-generation library of synthetic peptides. **a** In vitro activity of Pol-CP-NH_2_ and second-generation of analogs against Gram-positive bacteria (*Micrococcus luteus, Staphylococcus aureus, Staphylococcus epidermidis* and *Bacillus megaterium*), Fungi (*Candida albicans* and *Candida tropicalis*) and Gram-negative bacteria (*Escherichia coli, Enterobacter cloacae* and *Serratia marcescens*). Assays were performed in three independent replicates and heat map OD_600_ values are the arithmetic mean of the replicates in each condition. **b** MIC (µmol L^−1^) average vs. *f*_H_ in TFE/Water solution. **c** Graph correlating MIC (μmol L^−1^) averages vs. H and **d** MIC (μmol L^−1^) averages vs. μH, where blue boxes represent peptides with lower activity and red boxes show peptides with higher activity compared to the wild-type, in which we can observe ranges of optimal activity in determined intervals of *H* and μH values

Next, peptides were tested against a larger panel of Gram-positive and Gram-negative bacteria and two species of *Candida* (Fig. 5a). As anticipated by our previous structure-activity relationship analysis (Figs. 2–4), mutations made within the hydrophobic face led to decreased helical fraction values (Fig. 5b) and resulted in loss of antimicrobial activity (Fig. 5a). The hydrophobicity and hydrophobic moment functional hotpots identified previously (Fig. 2) also correlated with maximal antimicrobial activity in the sub-micromolar range (Fig. 5b–d). This behavior confirms the importance of the hydrophobic face of the peptide in both structure and activity, since we can observe clearly a helix-coil transition when peptides are in contact with membranes or membrane-like environments, such as the vesicles used in the circular dichroism experiments. The three most active AMPs, [Lys]^4^-Pol-CP-NH_2_, [Lys]^7^-Pol-CP-NH_2_ and [Gly]^1^-Pol-CP-NH_2_, were tested against the initial panel of bacteria (*E. coli* BL21, *P. aeruginosa* PA01 and PA14, and *S. aureus* ATCC12600—Supplementary Table 2). All peptides were active against *E. coli*, even at very low concentrations (<2 μmol L^−1^), and moderately active against *P. aeruginosa* PA01 (8 – 32 μmol L^-1^), with [Gly]^1^-Pol-CP-NH_2_ presenting surprisingly high activity against *P. aeruginosa* PA14 (<2 μmol L^−1^) (Supplementary Figure 5). The peptides, except for [Gly]^1^-Pol-CP-NH_2_ (64 μmol L^−1^), showed high activity against *S. aureus* (8–16 μmol L^−1^) (Supplementary Figure 5). Thus, synthetic peptides exhibited differential antimicrobial activity, which was predicted by physicochemical parameters.

Molecular dynamics simulations were performed in water and 60% TFE/water solution (v/v) (Supplementary Figure 6) for the three most active peptides ([Lys]^4^-Pol-CP-NH_2_, [Lys]^7^-Pol-CP-NH_2_, and [Gly]^1^-Pol-CP-NH_2_) (Fig. 5a) from the second generation library (Fig. 4a) and one of the least active analogs ([Lys]^5^-Pol-CP-NH_2_) (Fig. 5a). The simulations showed that the peptides were less structured in water than in the TFE/water solution. Differently from the Ala-scan results, in TFE/water medium, the introduction of a Lys residue in the hydrophobic face core ([Lys]^5^-Pol-CP-NH_2_) preserved the peptide structure (Supplementary Figure 6B), probably because of hydrophobic interactions provided by the longer aliphatic portion of the Lys side chain compared to the Ala side chain previously introduced. Substitutions made to the hydrophilic face led to stabilized helical structures (Supplementary Figure 6A), with increased helical content when compared to the wild-type peptide (Fig. 4c and Supplementary Figure 6B). On the other hand, the introduction of a Gly residue to the hydrophobic face of the peptide destabilized the N-terminus of the structure (Supplementary Figure 6A), as expected: Gly is known to increase flexibility and disfavor helical structure^36^ and is generally directly correlated with increased cytotoxic activity^52^.

**Fig. 6:**
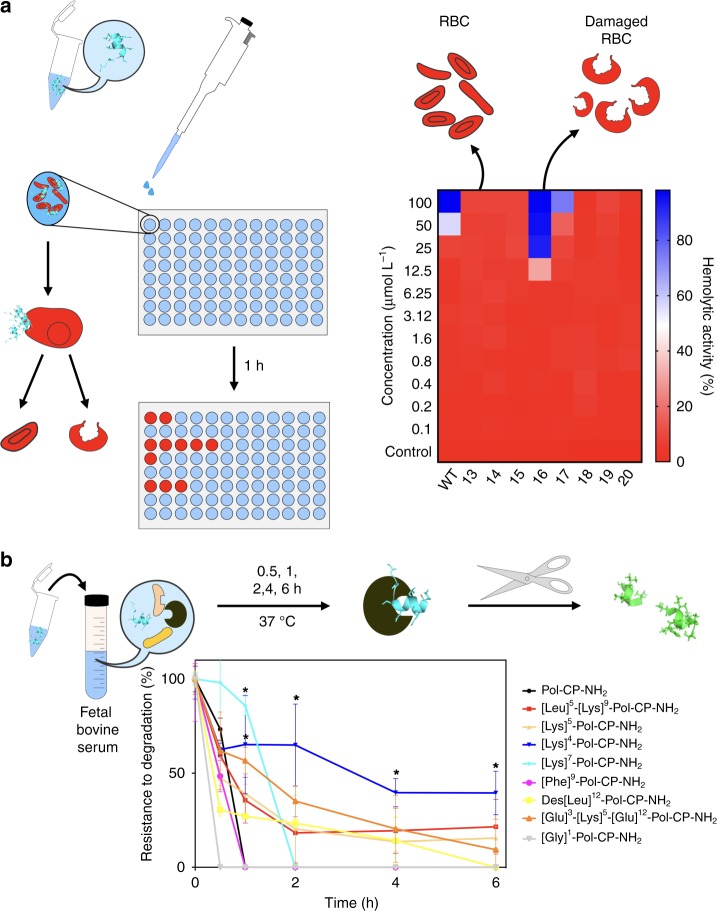
Hemolysis and resistance to protease-mediated degradation of engineered peptides. **a** Schematic of experimental design and hemolytic assay results of Pol-CP-NH_2_ and derivatives, where hemolytic activity was evaluated by incubating the peptides (0.1–100 μmol L^−1^) with human red blood cells in PBS at room temperature for 1 h. Experiments were performed in three independent replicates. **b** Resistance to degradation of Pol-CP-NH_2_ and analogs exposed to fetal bovine serum (FBS) proteases for 6 h. Experiments were done in three independent replicates (statistical significance was determined using one-way ANOVA, *p < 0.039, error bars represent standard deviation values)

The hemolytic activity of AMPs directly correlates with their interaction with zwitterionic membranes, which they subsequently lyse^53^. Tuning AMP features to modulate membrane interactions to minimize their effect on erythrocyte membranes while preserving activity against bacteria is a long-standing challenge in the field. One of the most important parameters to achieve this selectivity is tuning the electronic density of AMPs, which are usually positively charged and attracted to the negatively charged membranes of microorganisms, whereas eukaryotic cells display zwitterionic lipids in their membrane^54^. Mammalian cells present higher amounts of cholesterol in their membrane, which stabilizes the lipid bilayer by increasing cohesion and mechanical stiffness^55^, making it difficult for the membranes to bend and, consequently, to be permeabilized by AMPs. After the initial electrostatic interactions, the hydrophobic face of the amphipathic structure of AMPs interacts directly with the nonpolar region of the microorganism membrane, destabilizing it and leading to membrane disruption and cell death^56,57^. Our design methodology focused primarily in enhancing features that would increase peptides interaction with negatively charged membranes. Thus, the hemolytic activity (Fig. 6a) of the peptides was tested to verify their translatability prior to in vivo assays. Analog [Phe]^9^-Pol-CP-NH_2_ was as hemolytic as the wild-type peptide (between 50 and 100 µmol L^−1^). [Lys]^7^-Pol-CP-NH_2_ was the only analog with higher hemolytic activity than the wild-type (12.5 μmol L^−1^). None of the other analogs exhibited hemolytic activity in the range of concentrations evaluated (0–100 μmol L^−1^).

Stability is an issue often limiting the translation of AMPs into the clinic^58^. Pol-CP-NH_2_ is a natural occurring cationic AMP, and most cationic peptides are not stable in the presence of peptidases^59^. The stability of the second generation of Pol-CP-NH_2_ derivatives (Table 3) in fetal bovine serum was assessed (Fig. 6b). Most analogs were degraded in a few minutes after exposure to serum proteases, including [Gly]^1^-Pol-CP-NH_2_. However, [Lys]^7^-Pol-CP-NH_2_ and [Lys]^4^-Pol-CP-NH_2_ demonstrated increased resistance to protease-mediated degradation, particularly [Lys]^4^-Pol-CP-NH_2_, which persisted (~50% of initial concentration added) even after six hours of exposure (Fig. 6b). The introduction of Lys residues in both cases favored a higher helical stabilization compared to the other modifications made (Fig. 4; Table 4) and this is known as strategy to achieve higher resistance to degradation^60^. However, there are other elaborated approaches that could be used as potential stability enhancers, such as introducing restrictions (lactam and disulfide bridged peptides), cyclic peptides and/or introduction of lipids or carbohydrates as peptides conjugates^61^.

### Cytotoxicity and in vivo antimicrobial activity against *P*. *aeruginosa*

**Fig. 7:**
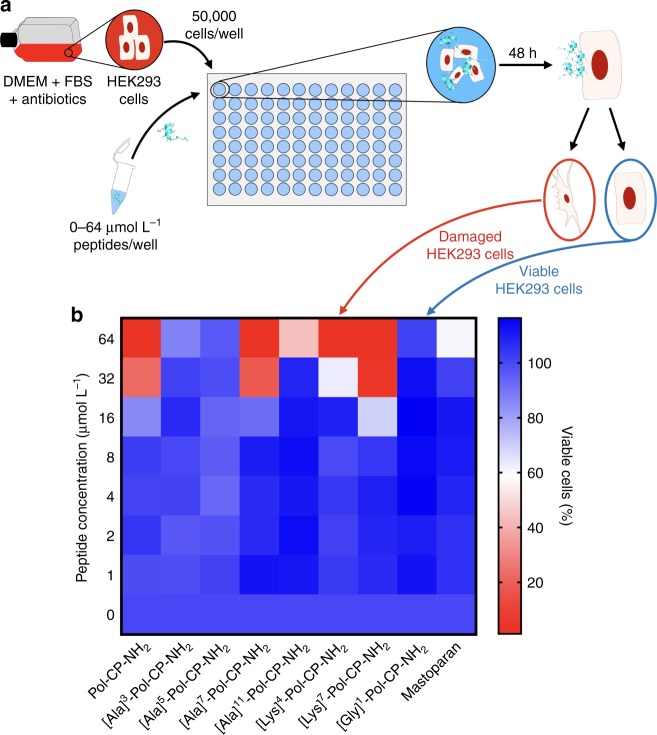
Cytotoxicity of engineered peptides. **a** Schematic of the experimental design for cytotoxicity assays of Pol-CP-NH_2_ and derivatives against HEK293 human embryonic kidney cells. Briefly, cells were cultured in DMEM medium supplemented with FBS and antibiotics at 37 ^o^C and 5% CO_2_ and **b** results obtained by seeding HEK293 50,000 cells and incubating with peptides’ solution (0–64 μmol L^−1^) at 37 ^o^C for 48 h. Cell viability was measured by MTS assay. All experiments were performed in independent triplicates (heat map values represent mean values of three independent replicates)

**Fig. 8:**
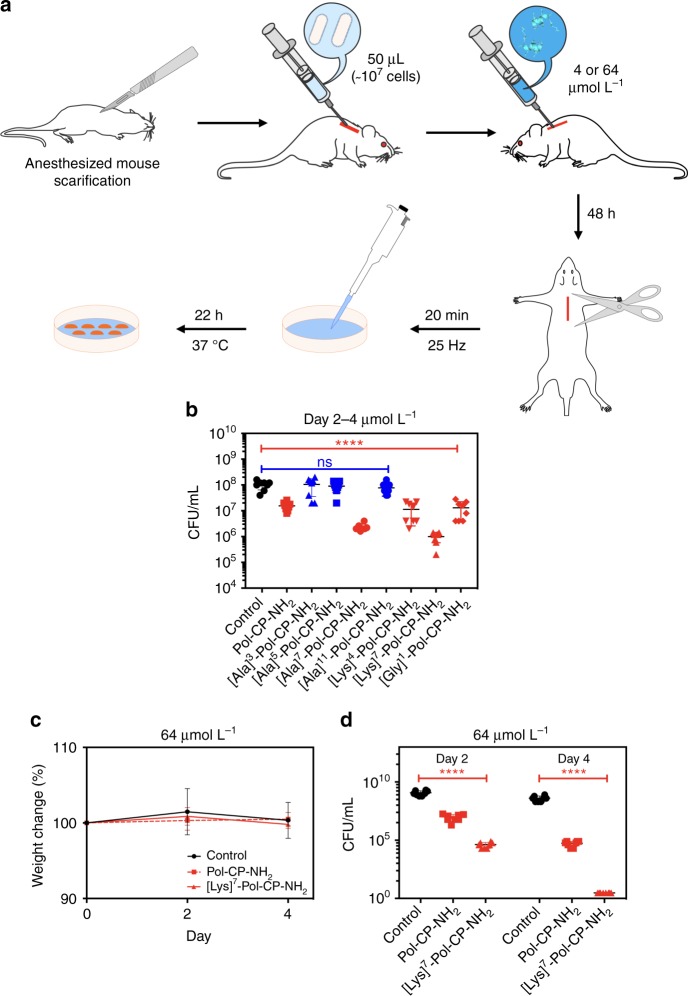
In vivo activity of Pol-CP-NH_2_ and its analogs. **a** Schematic of the experimental design. Briefly, the back of mice was shaved and an abrasion was generated to damage the stratum corneum and the upper layer of the epidermis. Subsequently, an aliquot of 50 μL containing 5 × 10^7^ CFU of *P. aeruginosa* in PBS was inoculated over each defined area. One day after the infection, peptides (4 μmol L^−1^) were administered to the infected area. Four animals per group were euthanized and the area of scarified skin was excised two days post-infection **b** homogenized using a bead beater for 20 min (25 Hz), and serially diluted for CFU quantification (statistical significance was determined using two-way ANOVA followed by Dunnett’s test, ****p < 0.0001). **c** Mouse body weight measurements throughout the experiment normalized by the body weight of non-infected mice. The wild-type peptide and the most active analog ([Lys]^7^-Pol-CP-NH_2_) were used at 64 μmol L^−1^, where infection and CFU quantification were performed as described in **b**, the body weight of mice treated with peptide did not change abruptly compared to untreated mice. **d** Longer experiment (four days) using a higher concentration (64 μmol L^−1^) of peptides Pol-CP-NH_2_ and [Lys]^7^-Pol-CP-NH_2_ (four mice per group and statistical significance was determined using two-way ANOVA followed by Dunnett’s test, ****p < 0.0001)

Several peptides from both generations identified as most active (i.e., antimicrobial hits) and least active (i.e., negative controls) against the Gram-negative bacterium *P. aeruginosa* (Figs. 2, 4) were tested for cytotoxicity against human embryonic kidney cells (HEK293) (Fig. 7). The wild-type peptide presented cytotoxic activity at a lower concentration (32 µmol L^−1^) than its MIC against *P. aeruginosa* (64 µmol L^−1^), whereas all synthetic analogs presented low cytotoxicity against HEK293 cells (Fig. 7b). The lead peptides ([Lys]^4^-Pol-CP-NH_2_ and [Lys]^7^-Pol-CP-NH_2_) exhibited certain cytotoxicity at concentrations two-fold to four-fold higher than their MICs against *P. aeruginosa* (Fig. 7b). The least active analogs were not cytotoxic in the range analyzed (0–64 µmol L^−1^) (Fig. 7b). Our lead peptide hit, [Lys]^7^-Pol-CP-NH_2_, displayed cytotoxicity at 16 µmol L^−1^; therefore, we used a nontoxic dose (4 µmol L^−1^) of this and the other lead peptides to assess their anti-infective potential in vivo using a scarification mouse model (Fig. 8).

A skin infection was induced in mice, after which a single dose of 4 µmol L^−1^ of peptides was administered (Fig. 8a). The antimicrobial activity of all peptides was consistent with results obtained in vitro (Figs. 2 and 5). The lead peptide derivatives, having substitutions in position 7 ([Ala]^7^-Pol-CP-NH_2_ and [Lys]^7^-Pol-CP-NH_2_), were the most active, and [Gly]^1^-Pol-CP-NH_2_ and [Lys]^4^-Pol-CP-NH_2_ demonstrated comparable activity to the wild-type peptide (Fig. 8b). A single dose of the lead peptide [Lys]^7^-Pol-CP-NH_2_, which was non-toxic to mice^62–65^ (Fig. 8c), further demonstrated anti-infective activity virtually sterilizing skin infections after four days (Fig. 8d).

## Discussion

AMPs represent promising alternatives to conventional antibiotics to combat the global health problem of antibiotic resistance^4,6^. However, their development is limited by the lack of methods for cost-effective^28,66,67^ and rational^68^ design. Although some alternative methods to overcome these limitations have been proposed^69–71^, we are far from understanding the structure-activity relationship of these agents, which would provide a more substantial basis for their rational design and accelerate their translation to the clinic.

Here, we describe a systematic structure–activity relationship design approach aimed at revealing the sequence requirements for antimicrobial activity of a natural wasp venom AMP^30^ and several of its derivatives. Through single-residue substitutions guided by identified physicochemical activity determinants, we generated peptide antibiotics with anti-infective potential in a mouse model.

Pol-CP-NH_2_ is a chemotactic peptide from the venom of a tropical species of wasp that presents 12 residues typical of peptides found in these wasp species^30^. Wasp venom peptides usually present characteristic motifs, such as a Phe-Leu-Pro tripeptide at the amino terminal side, which are thought to be responsible for their mechanism of action. Pol-CP-NH_2_, however, lacks these specific sequence patterns, which may explain its decreased antimicrobial activity compared to other wasp venom peptides such as mastoparan and VesCP^30,72^. Also unlike other wasp venom peptides, Pol-CP-NH_2_ lacks a central cationic Lys residue in its seventh position^72^. Pol-CP-NH_2_ does contain a Lys residue in its tenth position, like its analog protonectin. The main structural difference between protonectin and Pol-CP-NH_2_ is the replacement of the eleventh residue in protonectin (Gly) by a Ser residue in the Pol-CP-NH_2_ sequence. The differences between Pol-CP-NH_2_ and other mastoparan-like peptides does not prevent it from presenting chemotactic activity. Pol-CP-NH_2_ was described as cause of mast cell degranulation activity reduction, mast cell lysis, besides of inducing chemotaxis of polymorphonucleated leukocytes, characteristics usually observed for wasp venom mastoparan-like peptides^72^.

MIC (Fig. 2), CFU counts (Supplementary Figure 2), and circular dichroism spectra (Fig. 3) assays using Ala-scan analogs revealed that positions 3 (Gly), 4 (Thr), 6 (Leu), 7 (Gly), and 11 (Ser) were residues with side chains that did not substantially contribute to structure and function, whereas positions 5 (Ile) and 10 (Lys) were identified as key determinants of structure and antimicrobial function. Thus, the hydrophilic residues present in Pol-CP-NH_2_ (Fig. 2d) were not important for the peptide to adopt a helical structure or for antimicrobial function, with the exception of the only charged residue (Lys). On the other hand, the hydrophobic residues present in the wild-type peptide appear to be vital for peptide structure because of their aliphatic side chains and the hydrophobic interactions of these side chains, which enable the unstructured-to-helix transition in an environment, such as the bacterial membrane or TFE/water, that favors structuring of the peptide (Fig. 3; Table 2).

To test the importance of the hydrophilic residues and increased charge in structure-function, we engineered synthetic analogs. Two of these ([Lys]^4^-Pol-CP-NH_2_ and [Lys]^7^-Pol-CP-NH_2_), which had insertions in the hydrophilic face at positions that would keep the hydrophobicity and hydrophobic moment within the optimal range (Fig. 2b, c), impacted favorably both structure and antimicrobial activity (Figs. 2d and 3b). One of the analogs ([Lys]^5^-Pol-CP-NH_2_) showed decreased antimicrobial activity because a positive charged residue was inserted in the hydrophobic face leading to decreased hydrophobicity and hydrophobic moment. Results obtained with these analogs show that, even with the insertion of a charged residue, the position of the insertion and the overall structure are more important to antimicrobial activity than increased net positive charge, as described for other cationic amphipathic AMPs^73–75^.

The impact of the introduction of charge via the insertion of Lys residues in positions 4, 5, and 7 was also predicted in our initial experiments (Figs. 4 and 5). Increasing helical content led to increased antimicrobial activity against a larger set of Gram-positive and Gram-negative bacteria and fungi. When the insertion was made within the hydrophilic face, we observed enhanced antimicrobial activity; the opposite effect was obtained when the substitution was made within the hydrophobic face of the peptide.

To analyze the combined effect of charge and the importance of the residues’ side chains on the hydrophobic face, we synthesized other analogs with double ([Leu]^5^-[Lys]^9^-Pol-CP-NH_2_) and triple substitutions ([Glu]^3^-[Lys]^5^-[Glu]^12^-Pol-CP-NH_2_) based on two-dimensional helical wheels (Supplementary Figure 4). These modifications were predicted to change the physicochemical features as much as single mutations at those positions with slight changes in their side chain size, and a single substitution with an aromatic hydrophobic residue to increase hydrophobicity in the middle of the hydrophobic face of the amphipathic structure ([Phe]^9^-Pol-CP-NH_2_). The substitution was made in position 9 as this is the closest position to the center of the hydrophobic face that did not alter the structure when Leu was replaced by Ala (Fig. 2 and Supplementary Figure 1). The insertion of a Phe residue in position 9 led to increased predicted hydrophobic moment. This insertion was made to evaluate cytotoxicity effects, as aromatic residues are known for their cytotoxic propensity due to enhanced hydrophobic interactions with lipids^74^. In addition, we designed a Gly-substituted analog ([Gly]^1^-Pol-CP-NH_2_), as Gly is commonly the first residue in AMPs^36^, and deleted the last residue (Des[Leu]^12^-Pol-CP-NH_2_), which changed peptide size and hydrophilic/hydrophobic ratio.

Results obtained with the newly designed analogs (Fig. 4) confirmed the hydrophobicity and hydrophobic moment optimal ranges observed previously (Fig. 2), although some exceptions were identified (Fig. 5c, d). Increasing the helical content consistently led to improved antimicrobial activity (Fig. 5b) in line with our previous data (Table 2). Collectively, tuning the helical content and net positive charge in specific positions (hydrophilic face) within the wild-type peptide enhanced its antimicrobial activity more predictably than modulating hydrophobicity.

A critical design property of AMPs is ensuring their specificity towards microorganisms, while minimizing unwanted toxicity against human cells. To check the toxicity of the second-generation of Pol-CP-NH_2_ derivatives, we performed assays using red blood cells either untreated or exposed to peptides (0–100 μmol L^−1^—Fig. 6a). Besides the wild-type, only the most active ([Lys]^7^-Pol-CP-NH_2_) and the most hydrophobic ([Phe]^9^-Pol-CP-NH_2_) analogs were hemolytic. The most active derivative, [Lys]^7^-Pol-CP-NH_2_, was hemolytic at 12.5 μmol L^−1^, a concentration substantially higher than its MIC against all the microorganisms tested (Figs. 5, 6). However, [Phe]^9^-Pol-CP-NH_2_ was as hemolytic as the wild-type (Fig. 6a) at doses corresponding to its average MIC (~50 μmol L^−1^) (Fig. 5a). The selectivity index (SI) of the hemolytic peptides was calculated as the ratio between the concentrations leading to 50% lysis of human erythrocytes and the average of the minimum concentration inhibiting bacterial growth of twelve different strains (SI = HC_50_/MIC)^61^, indicating how selective were the peptides. The most active analog, [Lys]^7^-Pol-CP-NH_2_, presented a SI of 9.2, which was greater than the one presented by the analog [Phe]^9^-Pol-CP-NH_2_ (2.5) and the wild-type (3.1). Indicating that even hemolytic in lower concentrations, [Lys]^7^-Pol-CP-NH_2_ was the most selective peptide towards a large variety of microorganisms including Gram-positive bacteria, Gram-negative bacteria and fungi, due to its higher antimicrobial activity. To further assess the toxicity profile of our peptides, we subjected lead compounds to cytotoxicity assays using HEK293 cells (human embryonic kidney cells). The cells were exposed to increasing doses of peptides (0–64 μmol L^−1^—Fig. 7), and cytotoxicity correlated with increased helical content.

The presence of charged residues on cationic amphipathic AMPs usually correlates with susceptibility to degradation by proteases. Being unstructured in water or saline media, these AMPs are easily cleaved by peptidases. We checked the stability of Pol-CP-NH_2_ and analogs in fetal bovine serum for six hours and observed a small difference in their resistance to degradation (Fig. 6b). The most resistant peptides were those with higher helical content.

Among the microorganisms studied, *P. aeruginosa* is a pathogenic Gram-negative bacterium responsible for pneumonia^76^ and for infections of the urinary tract^77^, gastrointestinal tissue^78^, skin and soft tissues^79–81^ and is very common in patients with cystic fibrosis^82^. Like other bacteria, *P. aeruginosa* is becoming resistant to common antibiotics^82^, and AMPs have been proposed as an alternative treatment to combat such infections^83^.

The skin infection mouse model used here involved inducing a *P. aeruginosa* infection and treating mice with a single dose of the selected peptides at low concentrations (4 μmol L^−1^) that did not induce hemolysis (Fig. 6) or cytotoxicity (Fig. 7). The effect of peptides on bacterial load in the infection site was assessed (Fig. 8). The analogs used in these assays were some of the lead peptides, e.g., peptides with high activity against *P. aeruginosa* (Fig. 2 and Supplementary Figure 5—[Ala]^7^-Pol-CP-NH_2_, [Ala]^11^-Pol-CP-NH_2_, [Lys]^4^-Pol-CP-NH_2_, [Lys]^7^-Pol-CP-NH_2_ and [Gly]^1^-Pol-CP-NH_2_,) and some less active analogs (Fig. 2a—[Ala]^3^-Pol-CP-NH_2_ and [Ala]^5^-Pol-CP-NH_2_), in addition to the wild-type. The antimicrobial activity observed in vivo (Fig. 8b) correlated with that obtained in vitro (Figs. 2, 5). The most active AMPs from the second-generation library had +3 net positive charge and exhibited superior activity compared to the wild-type and the Ala-scan active analogs. As expected, the peptides used as negative controls ([Ala]^3^-Pol-CP-NH_2_ and [Ala]^5^-Pol-CP-NH_2_) (Fig. 2) did not kill bacteria in vivo (Fig. 8b). [Ala]^11^-Pol-CP-NH_2_ was not active at the concentration tested (4 μmol L^−1^), which is not entirely surprising as its MIC value against *P. aeruginosa* is 4-fold higher (16 μmol L^−1^—Fig. 2a).

To show the suitability of our lead peptide [Lys]^7^-Pol-CP-NH_2_ as a peptide antibiotic, we tested its anti-infective activity against *P. aeruginosa* using our mouse model (Fig. 8a). As we showed that at 64 μmol L^-1^ the wild-type and [Lys]^7^-Pol-CP-NH_2_ were toxic, experiments were conducted, and animals were carefully monitored for any signs of toxicity. The lack of toxicity was confirmed by body weight measurements of the mice (Fig. 8c). Peptide treatment nearly sterilized the infection (Fig. 8d), thereby demonstrating the potential of this synthetic peptide as a novel antimicrobial.

We demonstrate physicochemical feature-guided design of AMPs as a useful tool for identifying functional determinants and designing novel synthetic peptide antibiotics. Using such an approach (Ala-scan and residue probability in determined positions), we have turned a naturally occurring AMP with lower activity against Gram-negative bacteria^30^, into potent variants capable of killing bacteria at nanomolar doses and displaying anti-infective activity in an animal models. Our study is an example of how to design small cationic amphipathic peptides to optimize biological activities and selectivity. We envision that the principles and approaches exploited here can be applied to other structure-activity studies in order to rationalize and better understand the role of physicochemical features and which approaches fit better to each family of peptides.

## Methods

### Solid-phase peptide synthesis (SPPS), purification and analysis

Ala-scan analogs were acquired from Biopolymers (MIT) and the second-generation of peptides was synthesized on a peptide synthesizer (PS3—Sync Technologies) using the fluoromethyloxycarbonyl (Fmoc) strategy, Rink Amide resin, with a substitution degree of 0.52 mmol g^−1^. Deprotection, coupling and cleavage steps were previously described by Torres et al.^35^.

The crude lyophilized peptides were then purified by preparative reverse-phase high-performance liquid chromatography and characterized by liquid-chromatography electrospray-ionization mass spectrometry, conditions which have previously been described in detail by Torres et al.^34^.

### Circular dichroism spectroscopy

Circular dichroism experiments were performed on a J-815 Circular Dichroism Spectropolarimeter (Jasco). Measurements conditions are described by Torres et al.^84^ Peptides at 50 μmol L^−1^ were analyzed in ten different environments: water, PBS (pH 7.4), methanol/water (1:1; v-v), TFE/Water (2:3, 3:2 and 4:1; v-v), 10 mmol L^−1^ POPC, 10 mmol L^−1^ POPC:DOPE (3:1) and 10 mmol L^−1^ POPC:POPG (3:1). Vesicles were prepared according to Torres et al.^35^. A Fourier transform filter was applied to minimize background effects.

### Microorganisms

The following strains were used: *Micrococcus luteus* A270, *Staphylococcus aureus* ATCC29213, *Staphylococcus epidermidis* ATCC12228, *Bacillus megaterium* ATCC10778 *Escherichia coli* SBS 363, *Enterobacter cloacae* β-12, *Serratia marcescens* ATCC4112, *Candida albicans* MDM8, *Candida tropicalis* IOC4560 from Instituto Butanta, São Paulo, Brazil, and *Escherichia coli* BL21, *Pseudomonas aeruginosa* PA14, *Pseudomonas aeruginosa* PA01 and *Staphylococcus aureus* ATCC12600 from Synthetic Biology Group at MIT.

### MIC assays

The MIC assays were performed using the broth microdilution method^85,86^. Peptides were added to the plate as solutions in BM2 minimal medium at concentrations ranging from 0 to 128 μmol L^−1^, and the bacteria were inoculated at 5 × 10^5^ CFU mL^-1^ per well. The plates were incubated at 37 °C for 24 h. The MIC was defined as the lowest concentration of compound at which no growth was observed. Additional liquid growth inhibition assays were done as described by Torres et al.^35^. All assays were done in three independent replicates.

### Bacterial killing experiments

Killing experiments involved performing 1:10,000 dilutions of overnight cultures of *E. coli* BL21, *S. aureus* ATCC12600, *P. aeruginosa* PA01 and PA14 in the absence or presence of increasing concentrations of Pol-CP-NH_2_ derivatives (0–64 μmol L^−1^). After 24 h of treatment, 10-fold serial dilutions were performed, bacteria were plated on LB agar plates (*E. coli* BL21 and *S. aureus* ATCC12600) and Pseudomonas Isolation Agar (*P. aeruginosa* PA01 and PA14) and allowed to grow overnight at 37 ^o^C after which colony forming unit (CFU) counts were recorded, according to Wiegand et al.^85^.

### Hemolytic activity assays

The activity of peptides against human erythrocytes was performed according to Torres et al.^35^. Human red blood cells were obtained from healthy donors at Hospital Vital Brasil in accordance with Instituto Butantan Ethical Guidelines (protocol CEUAIB #I-1345/15). Briefly, hemolysis was determined by reading the absorbance at 405 nm of each well in a bed of plates. One percent SDS in PBS solution was used as positive control^87,88^ and PBS only was used as a negative control. MHC was defined as the maximal non-hemolytic concentration.

### Stability assays

The resistance to degradation assay is detailed by Powell et al.^89^, and peptides were exposed to GIBCO fetal bovine serum. The reaction kinetics were followed by reverse-phase liquid chromatography and the percentage of remaining peptide was calculated by integrating the peptide peak area.

### Cytotoxicity assays

Human embryonic kidney 293 (HEK 293) cells were cultured in Dulbecco’s Modified Eagle Medium suplemented with 10% Fetal Bovine Serum and 1% penicillin-streptomycin at 37 ^o^C in 5% CO_2_. The day before treatment, 50,000 HEK 293 cells were seeded into each well in 96-well plates. The peptides were added at concentrations ranging from 0 to 64 μmol L^−1^ and 48 h after exposure, cell viability was measured by means of MTS (dimethylthiazol-carboxymethoxyphenyl-sulfophenyl-tetrazolium) assay. Experiments were performed in three independent replicates for each condition.

### Scarification skin infection mouse model

The anti-infective activity of peptides against *P. aeruginosa* strain PA14 in a mouse model was assessed according to Pane et al.^90^ CD-1 IGS female mice (six-weeks-old) were used and maintained in accordance with the Guide for the Care and Use of Laboratory Animals in an AAALAC-accredited facility. All procedures were approved by the MIT’s Institutional Animal Care and Use Committee (IACUC), protocol number 1016-064-19. Two independent experiments were performed with 4 mice per group in each condition. Statistical significance was assessed using a one-way ANOVA.

### Molecular modeling

Molecular modeling studies were carried out according to Cardoso et al.^91^ in four successive steps: selection of a template structure; alignment between the template and target sequences; construction of atomic coordinates; and validation of the lowest free energy theoretical models. Initially, Blastp was performed and a fragment from the structure of a methyltransferase (chain A) (PDB entry: 3SSM)^92^ was select as template, taking into account parameters such as identity, coverage and e-value. All target sequences were individually aligned to the template and further submitted to comparative modeling simulations on MODELLER v. 9.17^93^. A total of 100 models were generated for each peptide and ranked according to their free energy scores (DOPE score). The lowest free energy models for each peptide were validated regarding their stereochemistry and fold quality on PROCHECK^94^ and ProSA-web^95^ servers. Finally, the validated structures were visualized and analyzed using PyMOL v. 1.8 (The PyMOL Molecular Graphics System, Version 2.0 Schrödinger, LLC).

### Molecular dynamics

Molecular dynamics simulations were conducted in hydrophilic environment (water) and in a mixture of 60% TFE/water (v/v). The GROMOS 43a1 force field^41^ was used and the simulation and analysis performed using the computational package GROMACS 5.0.4^42^. As initial structures, the validated models obtained from molecular modeling simulations were immersed in cubic boxes containing single point charge water molecules. Simulations in 60% TFE were also performed in cubic boxes, the peptides immersed in single point charge water molecules, followed by the insertion of TFE molecules until the ideal concentration was reached. Chloride ions (Cl^-^) were also added to neutralize the system’s charge. Conditions and parameters used are described in detail by Cardoso et al.^91^ All simulations were programmed in triplicate.

## Electronic supplementary material

Supplementary Information

## Data Availability

All data generated or analyzed in this study are included in this published material and its Supplementary Information file. Raw excel files of all data are available from the corresponding authors or first author on reasonable request.
