# Supplementary material for: Structure-function-guided exploration of the antimicrobial peptide polybia-CP identifies activity determinants and generates synthetic therapeutic candidates
Source: Commun Biol. 2018 Dec 7;1:221. doi: 10.1038/s42003-018-0224-2 (PMC6286318; doi:10.1038/s42003-018-0224-2)
Supplement: Supplementary file 1 — Supplementary Information [file 42003_2018_224_MOESM1_ESM.pdf]

Supplementary Information: Structure-Function-Guided Exploration of the Antimicrobial Peptide Polybia-CP Identifies Activity Determinants and Generates Synthetic Therapeutic Candidates

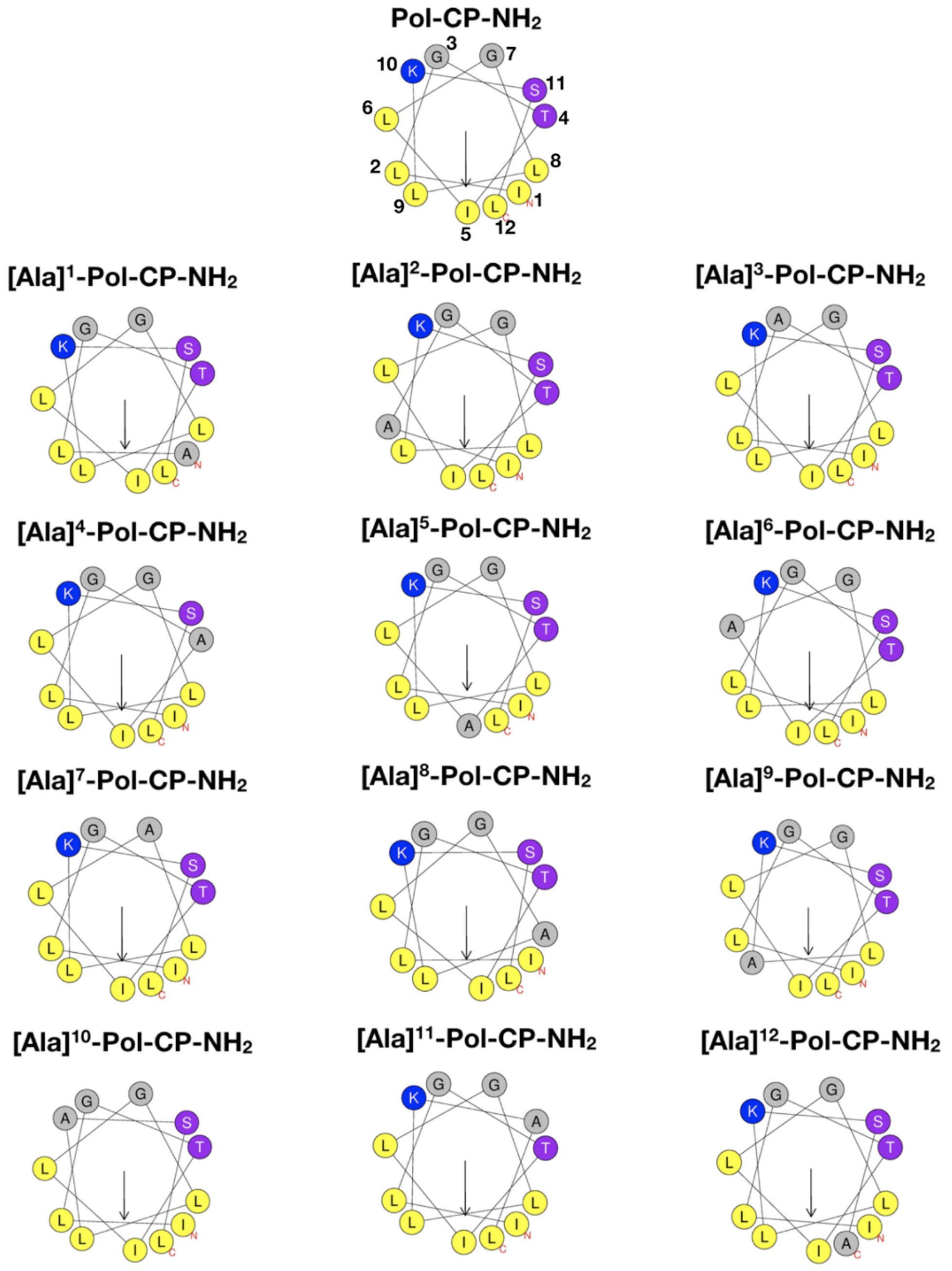

Supplementary Figure 1. Helical wheel representations of the Ala-scan Pol-CP-NH2 analogs generated using the Heliquest server considering theoretical helical structure and physicochemical properties derived from the amphipathic distribution. Yellow circles indicate hydrophobic/aliphatic residues, blue circles represent positively charged residues, purple circles indicate polar uncharged residues, and gray circles represent residues with hydrophobicity close to zero. The black arrows inside the helical wheel projection of each peptide represent their hydrophobic moment vector, whose magnitude is indicated by the size of the arrows.

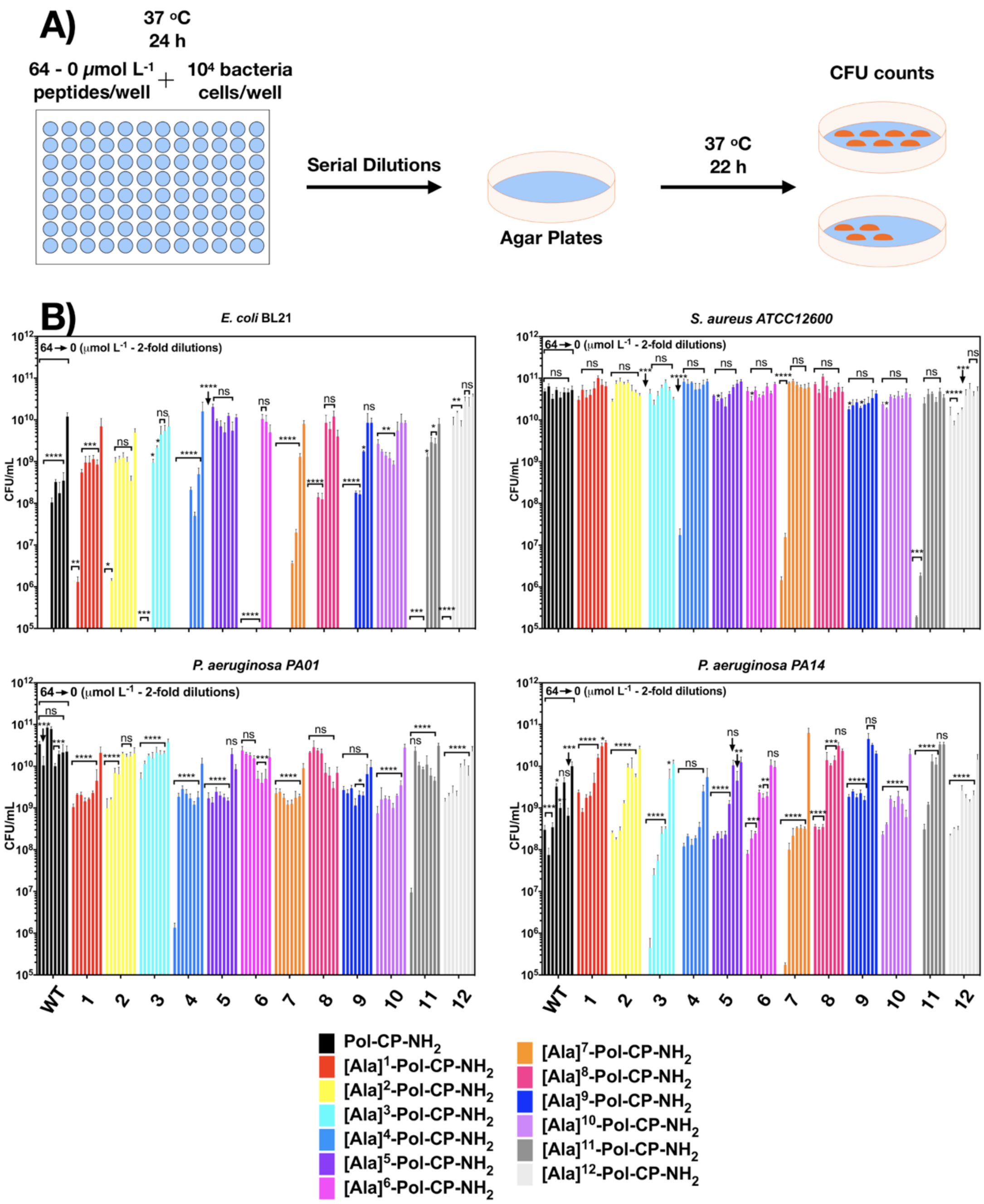

Supplementary Figure 2. (A) Schematic of the in vitro CFU count setup to assess antimicrobial activity of Pol-CP-NH_2 and Ala-scan analogs. Briefly, 10^4 bacterial cells and serially diluted (0 – 64 μmol L^{-1}) peptides were added to a 96-well plate and incubated at 37 ºC. One day after the exposure, the solution in each well was 10-fold diluted seven times and the serial dilutions were plated in agar plates, which were incubated for 22 h at 37 ºC. **(B)** Next, bacterial colonies were counted. All assays were performed in independent triplicates (statistical significance was determined using two-way ANOVA followed by Dunnett's test, error bars = standard error of the mean, ns = statistically not significant, $^{*}p<0.05$, $^{**}p<0.005$, $^{***}p<0.001$, $^{****}p<0.0001$).

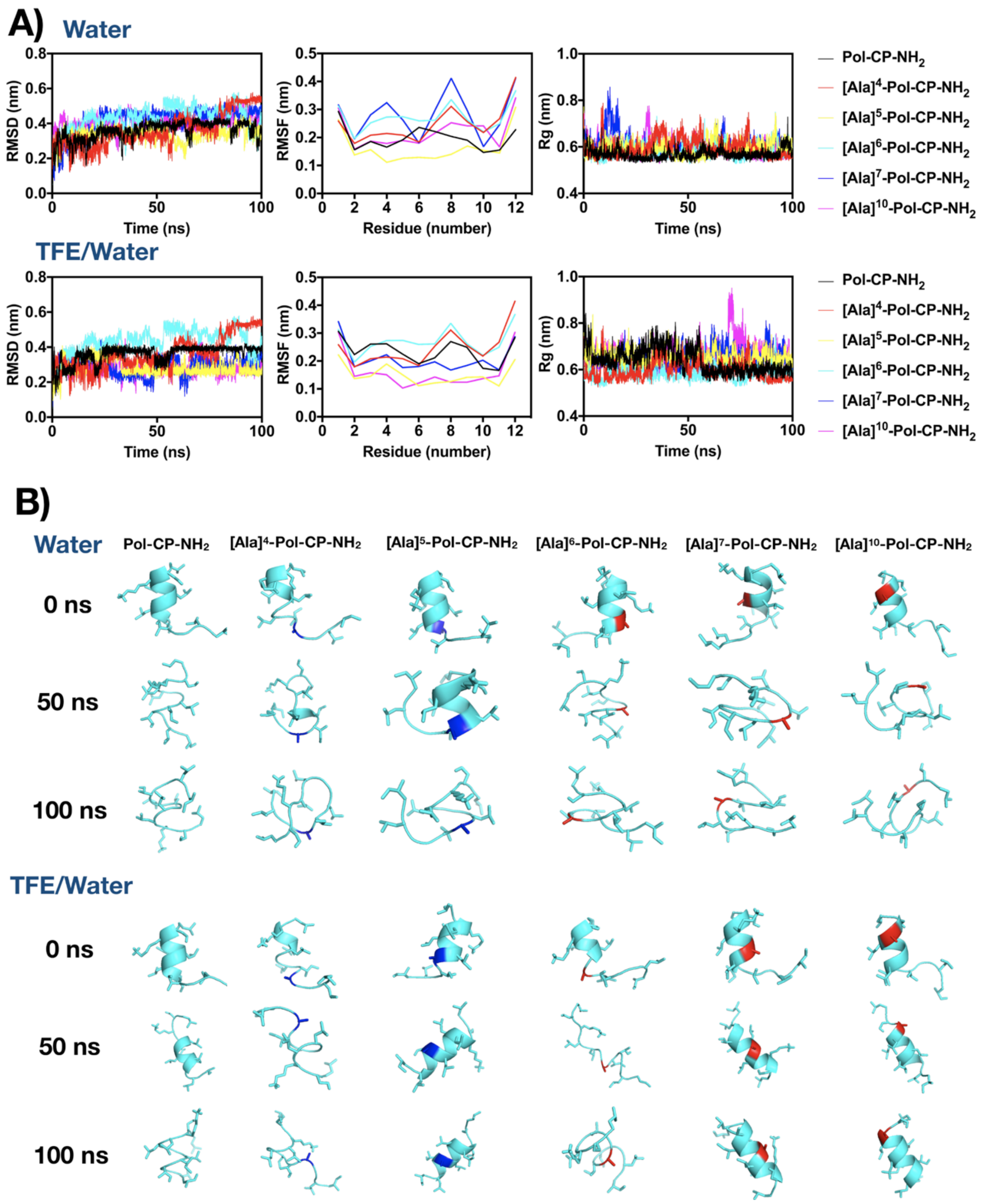

Supplementary Figure 3. (A) Graphical representation of residues movement of Pol-CP-NH_2 and Ala-scan analogs from molecular dynamics simulations in water and TFE/water (3:2, v:v), yielding root mean square deviation, root mean square fluctuation and radius of gyration after 100 ns. **(B)** Three-dimensional theoretical structures snapshots of Pol-CP-NH_2 and Ala-scan derivatives during 100 ns of molecular dynamics simulation. N-terminus of each peptide is always at the bottom.

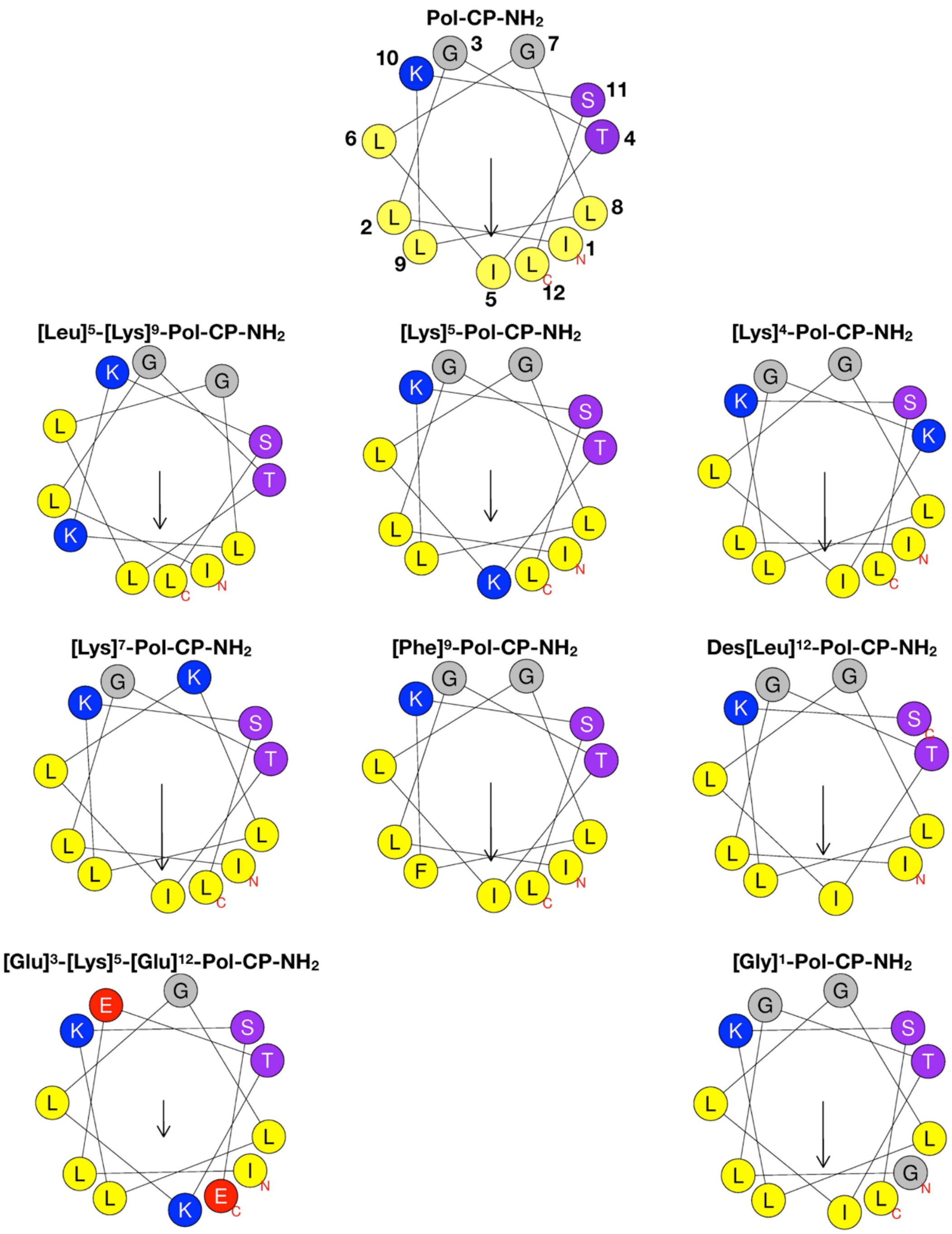

Supplementary Figure 4. Helical wheel representations of the second generation of Pol-CP-NH2 analogs generated using the Heliquest server considering theoretical helical structure and physicochemical properties derived from the amphipathic distribution. Yellow circles indicate hydrophobic/aliphatic residues, blue circles represent positively charged residues, purple circles indicate polar uncharged residues, red circles represent negatively charged residues, and gray circles represent residues with hydrophobicity close to zero. The black arrows inside the helical wheel projection of each peptide represent their hydrophobic moment vector, whose magnitude is indicated by the size of the arrows.

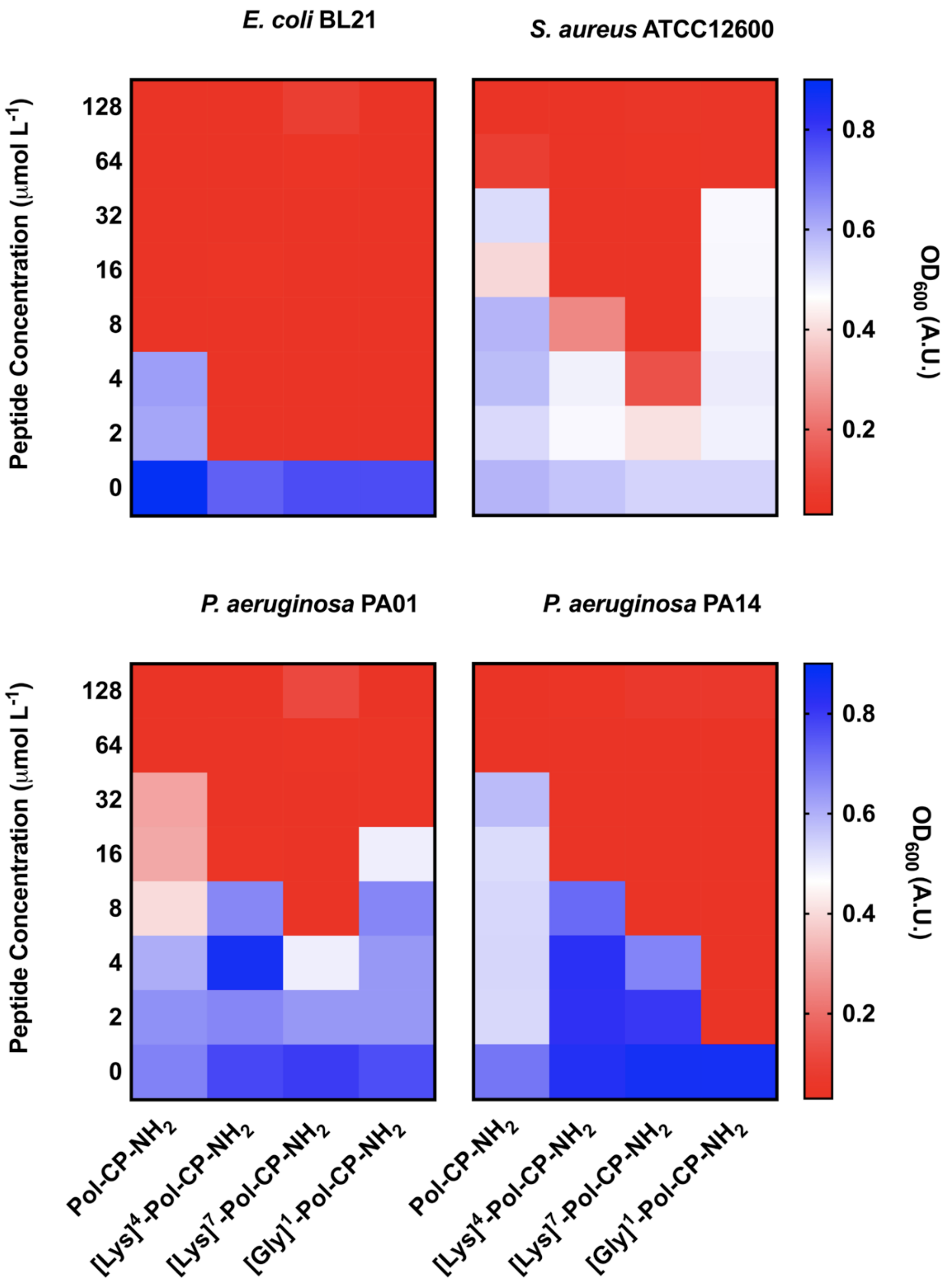

Supplementary Figure 5. In vitro antimicrobial activity of the lead peptides from the second generation of Pol-CP-NH_2 derived agents. Serially diluted (0 – 128 µmol L^{-1}) peptides were added to a 96-well plate containing 10^4 bacterial cells in each well and incubated at 37 °C for 24 h. After the exposure, the solution in each well was measured in a microplate reader (600 nm) to check inhibition of bacteria compared to the untreated controls and presented as heat maps of antimicrobial activities (µmol L^{-1}) against four bacteria strains: *Escherichia coli* strain BL21, *S. aureus* strain ATCC12600 and *P. aeruginosa* strains PA01 and PA14. Assays were performed in independent triplicates.

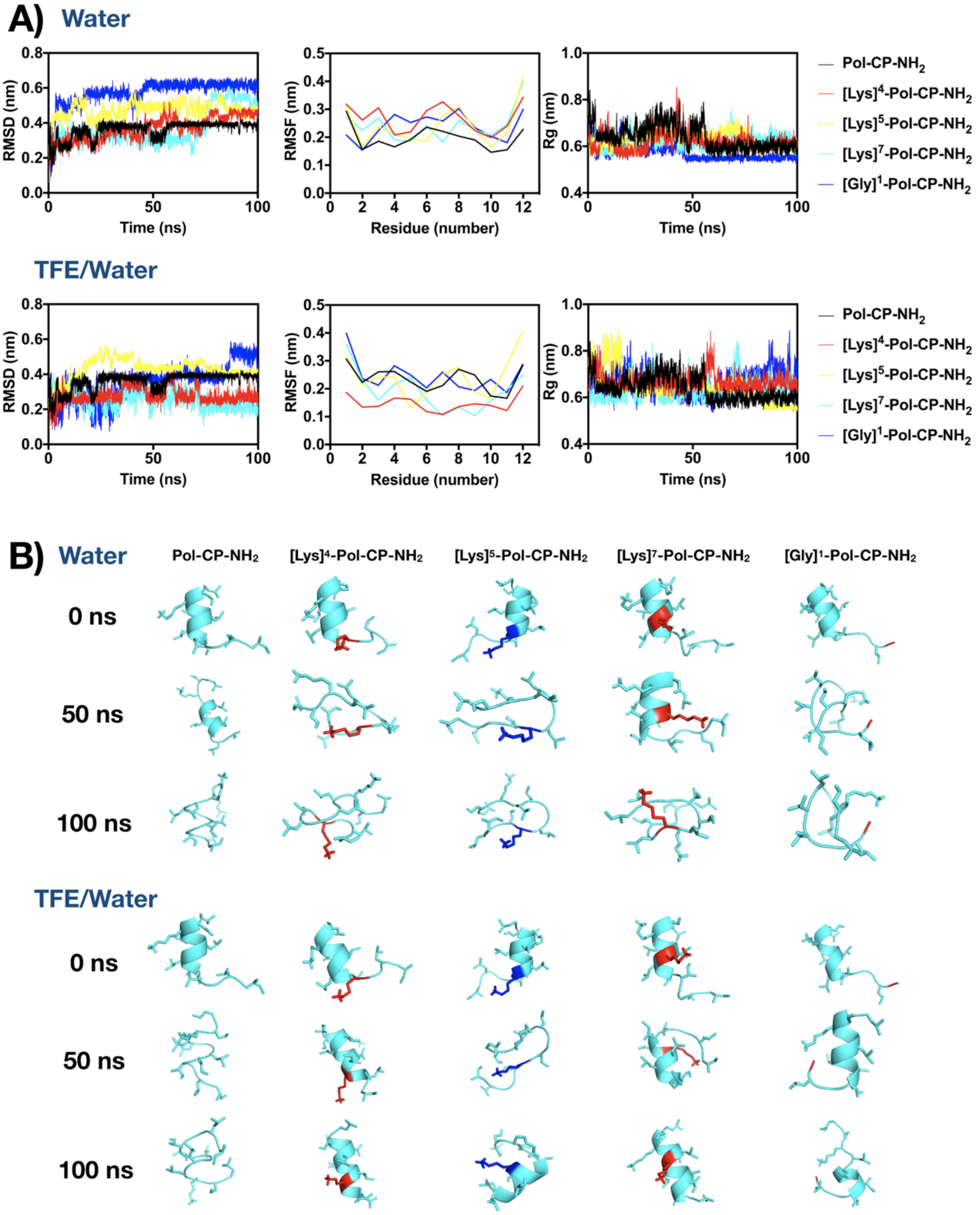

Supplementary Figure 6. (A) Graphical representation of residues movement of Pol-CP-NH2 and second generation of analogs from molecular dynamics simulations in water and TFE/water (3:2, v:v), yielding root mean square deviation, root mean square fluctuation and radius of gyration after 100 ns. **(B)** Three-dimensional theoretical structures snapshots of Pol-CP-NH2 and derivatives during 100 ns of molecular dynamics simulation. N-terminus of each peptide is always at the bottom.

Supplementary Table 1. Summary of Pol-CP-NH_2 and designed analogs.

Label	Peptide	Sequence	Molecular Weight (Da)	Observed Molecular Weight (Da)[a]	Purity (%)[b]	HPLC Retention Time (min)[c]	HC_{50} (μmol L^{-1})[d]	MIC Average (μmol L^{-1})	SI[e]	Cytotoxicity (μmol L^{-1})
**WT**	**Pol-CP-NH_2**	**ILGTILGLLKSL-NH_2**	**1239.8**	**1240.9**	**99**	**16.5**	**50.0**	**16.2**	**3.1**	**32.0**
1	[Ala]^1-Pol-CP-NH_2	**A**LGTILGLLKSL-NH_2	1197.8	1198.7	95	15.8	-	-	-	-
2	[Ala]^2-Pol-CP-NH_2	I**A**GTILGLLKSL-NH_2	1197.8	1198.8	96	15.4	-	-	-	-
3	[Ala]^3-Pol-CP-NH_2	IL**A**TILGLLKSL-NH_2	1253.8	1254.8	96	16.8	-	-	-	>64.0
4	[Ala]^4-Pol-CP-NH_2	ILG**A**ILGLLKSL-NH_2	1209.8	1210.8	93	16.8	-	-	-	-
5	[Ala]^5-Pol-CP-NH_2	ILGT**A**LGLLKSL-NH_2	1197.8	1198.8	94	14.4	-	-	-	>64.0
6	[Ala]^6-Pol-CP-NH_2	ILGTI**A**GLLKSL-NH_2	1197.8	1198.8	93	15.0	-	-	-	-
7	[Ala]^7-Pol-CP-NH_2	ILGTIL**A**LLKSL-NH_2	1253.8	1254.8	92	16.8	-	-	-	32.0
8	[Ala]^8-Pol-CP-NH_2	ILGTILG**A**LKSL-NH_2	1197.8	1198.8	93	15.0	-	-	-	-
9	[Ala]^9-Pol-CP-NH_2	ILGTILGL**A**KSL-NH_2	1197.8	1198.8	90	14.5	-	-	-	-
10	[Ala]^{10}-Pol-CP-NH_2	ILGTILGLL**A**SL-NH_2	1182.8	1183.8	92	17.5	-	-	-	-
11	[Ala]^{11}-Pol-CP-NH_2	ILGTILGLLK**A**L-NH_2	1223.8	1224.7	95	16.7	-	-	-	64.0
12	[Ala]^{12}-Pol-CP-NH_2	ILGTILGLLKS**A**-NH_2	1197.8	1198.8	92	14.6	-	-	-	-
13	[Leu]^5-[Lys]^9-Pol-CP-NH_2	ILGT**L**LGL**K**KSL-NH_2	1254.8	1256.0	99	11.5	>100.0	>50.0	-	-
14	[Lys]^5-Pol-CP-NH_2	ILGT**K**LGLLKSL-NH_2	1254.8	1255.9	99	11.5	>100.0	>50.0	-	-
15	[Lys]^4-Pol-CP-NH_2	ILG**K**ILGLLKSL-NH_2	1265.8	1266.8	98	15.0	>100.0	3.3	-	32.0
16	[Lys]^7-Pol-CP-NH_2	ILGTIL**K**LLKSL-NH_2	1309.8	1310.9	99	16.0	12.5	1.4	9.2	16.0
17	[Phe]^9-Pol-CP-NH_2	ILGTILGL**F**KSL-NH_2	1272.8	1274.0	99	15.7	50.0	20.0	2.5	-
18	Des[Leu]^{12}-Pol-CP-NH_2	ILGTILGLLKS-NH_2	1125.8	1126.8	99	13.4	>100.0	>50.0	-	-
19	[Glu]^3-[Lys]^5-[Glu]^{12}-Pol-CP-NH_2	IL**E**T**K**LGLLKS**E**-NH_2	1341.8	1341.8	99	9.2	>100.0	>50.0	-	-
20	[Gly]^1-Pol-CP-NH_2	**G**LGTILGLLKSL-NH_2	1182.8	1183.8	99	15.5	>100.0	16.7	-	>64.0

[a]LC/ESI-MS data were obtained on a Model 6130 Infinity mass spectrometer coupled to a Model 1260 HPLC system (Agilent), using a Phenomenex Gemini C18 column (2.0 mm × 150 mm, 3.0 μm particles, 110 Å pores). Solvent A was 0.1% TFA in water, and solvent B was 90% ACN in solvent A. Elution with a 5–95% B gradient was performed over 20 min, 0,2 mL min^{-1} flow and peptides were detected at 220 nm. Mass measurements were performed in a positive mode with the following conditions: mass range between 100 to 2500 m/z, ion energy of 5.0 V, nitrogen gas flow of 12 L min^{-1}, solvent heater of 250 °C, multiplier of 1.0, capillary of 3.0 kV and cone voltage of 35 V.

[b,c]HPLC profiles were obtained under the following conditions: Column Supelcosil C18 (4.6 x 150 mm), 60 Å, 5 μm; Solvent System: A (0.1% TFA/H_2O) and B (0.1% TFA in 90% ACN/H_2O); Gradient: 5–95% B in 30 minutes; Flow: 1.0 mL min^{-1}; λ = 220 nm; Injection Volume: 50 μL and Sample Concentration: 1.0 mg mL^{-1}.

[d]Concentration needed for 50% hemolysis caused by RBC exposure to peptides.

[e]Selectivity Index = $HC_{50}/MIC_{average}$ indicating peptides selectivity when in the presence of human erythrocytes.

Supplementary Table 2. Considerations for each one of the second library analogs designed synthesized in this work to check the importance of different kinds of substitutions and how well can the optimal hotspots describe activity propensities.

Peptide	Design Considerations
**ILGTILGLLKSL-NH_2**	Wild-type
**ILGTLLGLKKSL-NH_2**	To check if a subtle change in the side chain of the residue at position 5 would alter the peptide biological behavior even with an enhanced net positive charge
**ILGTKLGLLKSL-NH_2**	To check the importance of Ile presence at position 5 even when substituted by a residue with an additional charge since it decreased when it was substituted by an Ala residue
**ILGKILGLLKSL-NH_2**	To check effect of charge introduction at the interface of hydrophobic and hydrophilic faces
**ILGTILKLLKSL-NH_2**	Lys is common residue in the 7th position of small wasp venom peptides and Gly substitution by an Ala residue improved activity
**ILGTILGLFKSL-NH_2**	To check the effect of increased hydrophobicity
**ILGTILGLLKS-NH_2**	To check the effect of increasing hydrophilic/hydrophobic ratio
**ILETKLGLLKSE-NH_2**	To check the effect of the introduction of negatively charged residues on the hydrophilic face
**GLGTILGLLKSL-NH_2**	Gly is the most common residue in the 1st residue of small cationic amphipathic peptides
